# Quantification of archaea-driven freshwater nitrification from single cell to ecosystem levels

**DOI:** 10.1038/s41396-022-01216-9

**Published:** 2022-03-08

**Authors:** Franziska Klotz, Katharina Kitzinger, David Kamanda Ngugi, Petra Büsing, Sten Littmann, Marcel M. M. Kuypers, Bernhard Schink, Michael Pester

**Affiliations:** 1 Department of Biology, University of Konstanz, Universitätsstrasse 10, Konstanz, D-78457 Germany; 2 Max Planck Institute for Marine Microbiology, Celsiusstrasse 1, D-28359 Bremen, Germany; 3 Leibniz Institute DSMZ – German Collection of Microorganisms and Cell Cultures GmbH, Inhoffenstr. 7B, D-38124 Braunschweig, Germany; 4 Technical University of Braunschweig, Institute for Microbiology, Spielmannstrasse 7, D-38106 Braunschweig, Germany

**Keywords:** Water microbiology, Limnology

## Abstract

Deep oligotrophic lakes sustain large populations of the class *Nitrososphaeria* (Thaumarchaeota) in their hypolimnion. They are thought to be the key ammonia oxidizers in this habitat, but their impact on N-cycling in lakes has rarely been quantified. We followed this archaeal population in one of Europe’s largest lakes, Lake Constance, for two consecutive years using metagenomics and metatranscriptomics combined with stable isotope-based activity measurements. An abundant (8–39% of picoplankton) and transcriptionally active archaeal ecotype dominated the nitrifying community. It represented a freshwater-specific species present in major inland water bodies, for which we propose the name “*Candidatus* Nitrosopumilus limneticus”. Its biomass corresponded to 12% of carbon stored in phytoplankton over the year´s cycle. *Ca*. N. limneticus populations incorporated significantly more ammonium than most other microorganisms in the hypolimnion and were driving potential ammonia oxidation rates of 6.0 ± 0.9 nmol l^‒1^ d^‒1^, corresponding to potential cell-specific rates of 0.21 ± 0.11 fmol cell^–1^ d^–1^. At the ecosystem level, this translates to a maximum capacity of archaea-driven nitrification of 1.76 × 10^9^ g N-ammonia per year or 11% of N-biomass produced annually by phytoplankton. We show that ammonia-oxidizing archaea play an equally important role in the nitrogen cycle of deep oligotrophic lakes as their counterparts in marine ecosystems.

## Introduction

Freshwater lakes are important drinking water reservoirs. To be suitable as drinking water and to prevent toxicity to fish, ammonia must not accumulate. Nitrification prevents an accumulation of ammonia and converts it to nitrate via nitrite, with ammonia oxidation generally being the rate-limiting step [1]. Although nitrification does not directly change the inventory of inorganic N in freshwater ecosystems, it represents a critical link between mineralization of organic N and its eventual loss as N_2_ to the atmosphere through denitrification or anaerobic ammonium oxidation [1]. The process of ammonia oxidation is catalyzed by three different microbial guilds. Two of these, the ammonia-oxidizing archaea (AOA) [2, 3] and the ammonia-oxidizing bacteria (AOB) [4], oxidize ammonia to nitrite and depend on nitrite-oxidizing bacteria (NOB) [5] to complete nitrification by further oxidizing nitrite to nitrate. The third guild oxidizes ammonia directly to nitrate and is therefore referred to as complete ammonia oxidizers (comammox) [6, 7].

In general, the ratio of AOA to AOB decreases with increasing trophic state of freshwater lakes, as AOB have a preference for increased inorganic nitrogen loading and AOA are sensitive to copper complexation by organic matter [8–14]. In contrast, comammox bacteria have been detected at very low abundances in lacustrine systems, if at all [15, 16]. In snapshot analyses of oligotrophic lakes, AOA typically outnumbered AOB, especially in the deep oxygenated hypolimnion, and constituted up to 19% of the archaeal and bacterial picoplankton [10, 15, 17–20]. These observations resemble the situation in marine ecosystems, where AOA account for up to 40% of all microorganisms in the deep sea and thus are estimated to be among the most numerous microorganisms on Earth [21, 22]. In contrast to marine ecosystems, the impact of planktonic freshwater AOA on N cycling in oligotrophic lakes, which are often important drinking water reservoirs, is severely understudied and yet to be quantified.

Lake Constance is an oligotrophic, fully oxygenated lake that provides drinking water to more than five million people [23]. Being the second largest lake by volume in Central Europe, it represents an important model habitat for limnological processes [24]. A year-round survey of Lake Constance waters established that a single 16S rRNA gene-ecotype of AOA, related to the genus *Nitrosopumilus* (class *Nitrososphaeria*, “Thaumarchaeota” [25]), constituted the largest nitrifier population along the depth profile throughout the year, with AOB being two orders of magnitude less abundant [16]. At the same time, comammox bacteria were below the detection limit as based on diagnostic PCR of their *amoA* gene [16], which encodes the structural subunit of the key enzyme for ammonia oxidation, ammonia monooxygenase. Here, we link the predominant AOA population in this oligotrophic lake to ammonia oxidation activity and quantify this important ecosystem service at the single-cell, population, and ecosystem levels.

## Material and methods

### Study area and sampling procedure

Lake Constance is a monomictic peri-alpine lake with a maximum depth of 251 m. Sampling was conducted at the long-term ecological research station of the University of Konstanz (47.75788° N, 9.12617° E), which is located in the northwestern branch of Upper Lake Constance with a maximum depth of around 140 m. Upper Lake Constance is oligotrophic and has a permanently oxygenated hypolimnion throughout the year. In this study, we refer to Upper Lake Constance as “Lake Constance” excluding the smaller, shallow, and mesotrophic Lower Lake Constance. Measurements of environmental parameters followed standard procedures as detailed in the Supplementary Text.

**Fig. 1:**
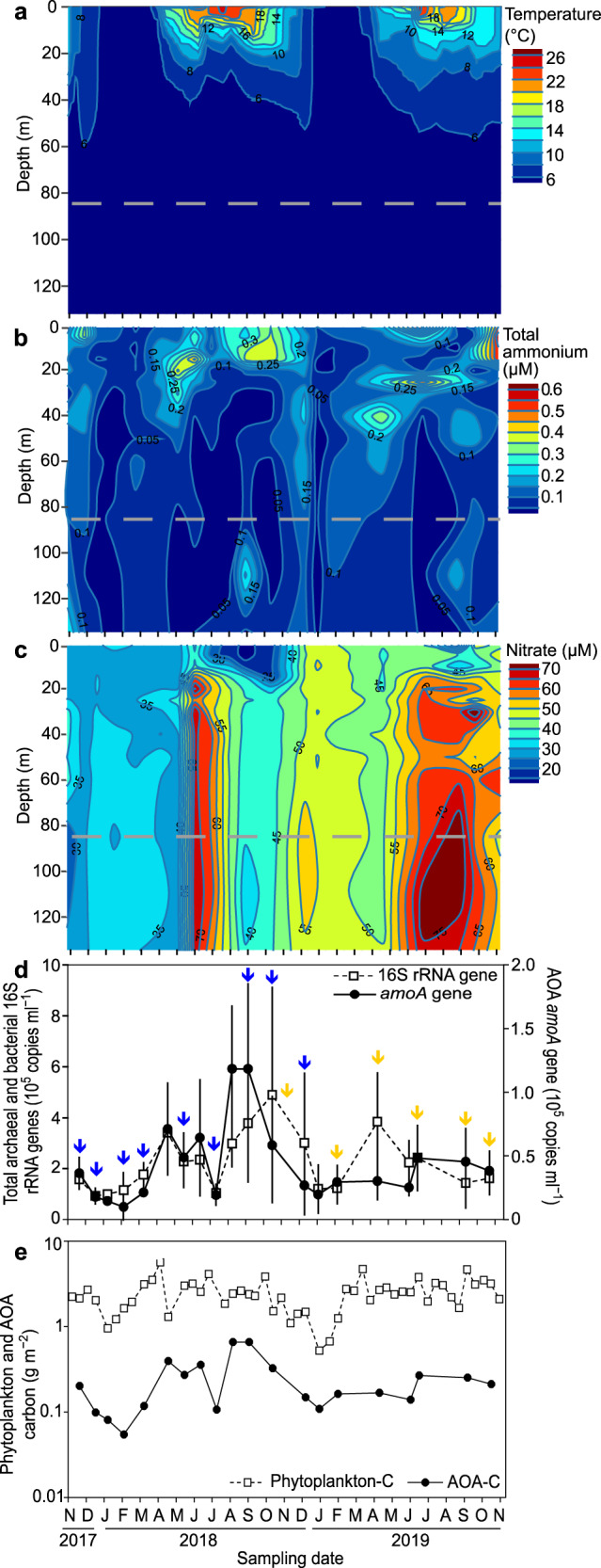
Temporal dynamics of the AOA population in relation to environmental parameters as well as the pico- and phytoplankton. Seasonality of physico-chemical parameters (**a**−**c**), the total archaeal and bacterial picoplankton as well as the AOA population at 85 m depth (**d**), and the carbon budget of the phytoplankton and AOA population (**e**) over two consecutive years in the water column of Lake Constance. Please note that the carbon budget values are shown on a logarithmic scale. The sampling depth for qPCR, metagenome and metatranscriptome analyses is indicated as a dashed gray line at 85 m. Arrows indicate time points for metagenome (blue) and metatranscriptome (yellow) sampling. qPCR analyses were done in replicates of 3 or 4, except for December 18^th^, 2018, where only duplicates could be measured for the AOA *amoA*.

For quantitative PCR, water was sampled from 85 m depth in three to four replicates (2.5–5.0 l) on a monthly basis from November 2017 to November 2019 [21.11.2017, 19.12.2017; 09.01.2018, 06.02.2018, 13.03.2018, 24.04.2018, 22.05.2018, 19.06.2018, 17.07.2018, 14.08.2018, 11.09.2018, 23.10.2018. 18.12.2018, 11.01.2019, 13.02.2018, 24.04.2019, 18.06.2019, 03.07.2019, 25.09.2019, 05.11.2019, (dd.mm.yyyy)]. Nine of these samples taken in the period from November 2017 to December 2018 were used for metagenome analyses as well (detailed in Fig. 1d and Supplementary Table S1). Water was pre-filtered through a 70 µm and 30 µm nylon mesh (Franz Eckert GmbH, Germany) to remove larger organisms and was filtered on board first through 5 µm and then through 0.2 µm polycarbonate filters (47 mm, Merck, Darmstadt, Germany) using pressurized air. Filters were stored immediately on dry ice on board and at –20°C in the laboratory until further processing.

Sampling for metatranscriptome analyses took place between November 2018 and November 2019 [07.11.2018, 13.02.2019, 24.04.2019, 03.07.2019, 25.09.2019, 05.11.2019 (dd.mm.yyyy)].Water was sampled in three replicates directly at the desired water depth of 85 m with a WTS-LV in-situ pump (McLane research laboratories, Inc., Massachusetts, USA). Within 1 h and depending on the biomass captured, 30.7–126.2 l of water (on average 67.2 ± 27.8 l) were filtered. Water was passed serially through a 30 µm mesh (Franz Eckert GmbH), 5 µm, and 0.22 µm filters (142 mm, Merck) with the initial flow rate set to 3.5 l min^−1^. Filters with 5 µm and 0.22 µm pore size were immediately placed on dry ice on board and stored at –80°C in the laboratory until further processing. Sampling of the third replicate in February and April 2019 failed.

### Nucleic acid extraction, qPCR, and CARD-FISH analyses

DNA and RNA were extracted separately from respective 0.22 µm filters after a modified protocol designed previously [26] as detailed in the Supplementary Text. Quantitative PCR assays of total bacterial and archaeal 16S rRNA genes as well as archaeal *amoA* were performed as described recently [16]. The *amoA* qPCR assay was specifically designed to target archaeal *amoA* retrieved from the water column of Lake Constance. The efficiency of qPCR assays targeting the archaeal *amoA* and the total bacterial and archaeal 16S rRNA genes were on average 72.5 ± 4.0% and 91.3 ± 1.6%, respectively. After each run, qPCR specificity was checked with a melting curve. In addition, qPCR products from selected runs were visualized on a 2.5%-agarose gel to verify the absence of unspecific PCR products. PCR-inhibitory substances were not evident by qPCR analyses of dilution series of two selected lake water DNA extracts.

For CARD-FISH analysis, 50 ml of lake water was sampled after 48 h (November 5th, 2019) or 67 h (June 18th, 2019) of the ^15^N-NH_4_^+^ incubation experiments described below and fixed overnight at 4°C with paraformaldehyde (final concentration 1%, without methanol, Electron Microscopy Sciences, Hatfield, PA, USA). Cells were filtered onto 0.2 µm polycarbonate filters (GTTP, Merck Millipore) and washed with filter-sterilized lake water. Filters were stored at −20°C until analysis. Before CARD-FISH, cells on filter sections were immobilized by embedding in 0.1% low-gelling agarose (MetaPhor, Lonza, Rockland, ME, USA). CARD-FISH was performed using a HRP-labeled oligonucleotide probe specific for *Nitrososphaeria*/”Thaumarchaeota” (HRP-labeled Thaum726 [GCTTTCATCCCTCACCGTC] and unlabeled competitors [Thaum726_compA: GCTTTCGTCCCTCACCGTC, Thaum726_compB: GCTTTCATCCCTCACTGTC]) [27, 28]. Negative controls using probe NonEUB [ACTCCTACGGGAGGCAGC] [29] were performed according to a defined protocol [30] to exclude unspecific signals. Further CARD-FISH analysis was done as described recently [31] and in the Supplementary Text.

### Metagenomics and metatranscriptomics

Metagenome sequencing libraries were prepared with the NEBNext Ultra DNA Library Prep Kit for Illumina (New England Biolabs GmbH, Frankfurt am Main, Germany) and sequenced on a NextSeq500 sequencer (Illumina) using 2 × 150 bases. In addition, DNA from November 2017 was sequenced by PacBio sequencing on a Sequel instrument (Pacific Biosciences, Menlo Park, CA, USA) using circular consensus sequencing with a target length of 2 kilobases. Bioinformatics processing, genome binning, and phylogenetic analysis followed standard procedures as described in the Supplementary Text.

For metatranscriptome sequencing, messenger RNA (mRNA) was enriched from total RNA extracts by depleting ribosomal RNA with the Ribo-off rRNA Depletion Kit for bacteria (Vazyme, Nanjing, China). Thereafter, the sequencing library was prepared with the TruSeq Stranded mRNA Library Prep (Illumina) and sequenced on a NextSeq500 sequencer using 2 × 150 bases. Bioinformatics processing to determine the transcriptional levels of selected genes as well as network analysis of co-transcribed genes are detailed in the Supplementary Text.

### Potential ammonia oxidation rate measurements

To determine potential ammonia oxidation and ammonium assimilation rates, ^15^N-NH_4_^+^-tracer experiments were carried out as described recently [31]. Water was sampled from 85 m depth using a Niskin bottle on 18.06.2019, 29.07.2019, 28.08.2019, and 05.11.2019 (dd.mm.yyyy). Thereafter, 4.5 l were distributed on board to 5-l glass bottles. Bottles were sealed with oxalic acid and sodium hydroxide cleaned butyl rubber stoppers and wrapped in aluminum foil to protect them from light. Within 1−7 h after sampling, ^15^N-tracer experiments were started by addition of (^15^NH_4_)_2_SO_4_ (10 µM final ^15^N-concentration) and bottles were incubated at in-situ temperature (4°C) in the dark. All incubations were done in biological triplicates. Subsamples of 10 ml were taken for potential ammonia oxidation rate measurements over a time period of 48 h (67 h in June), filter-sterilized (0.2 µm) and frozen at −20°C. ^15^N-ammonium labeling percentage was inferred in two steps: First, in-situ ammonium concentrations were determined using the spectrophotometric salicylate method [32]. Second, added ^15^N-ammonium was measured in samples taken immediately after isotope addition by conversion to N_2_ using alkaline hypobromite as described previously [33]. The amount of added ^15^N-ammonium was divided by the sum of in-situ ammonium and added ^15^N-ammonium to determine the labeling percentage, which was >99% for all samples. Thereafter, potential ammonia oxidation rates were determined from combined increase in ^15^N-nitrite and ^15^N-nitrate over time. First, ^15^N-nitrite was measured by conversion to N_2_ with sulfamic acid [34], and the resulting ^29^N_2_ was measured by gas chromatography isotope ratio mass spectrometry (IRMS) using an Isoprime Trace Gas for cryogenic concentration of gases coupled to a sector field IsoPrime100 with a multicollector for simultaneous detection of multiple masses (Isoprime, Manchester, UK). After the ^15^N-nitrite measurement, nitrate was reduced to nitrite using spongy cadmium and subsequently converted to N_2_ via sulfamic acid [34, 35]. To calculate potential ammonia oxidation rates, the sum of ^15^N-nitrite and ^15^N-nitrate was used to give the total ^15^NO_x_ production per time point. Rates were inferred from the slopes of linear regressions; only slopes that were significantly different from zero are reported (*P* < 0.05, one-tailed *t*-distribution test).

### Single cell ^15^N-uptake measurements from combined CARD-FISH nanoSIMS

After 48 h of the ^15^N-NH_4_^+^-tracer incubation experiment in November 2019, water samples were taken for *Nitrososphaeria*-specific CARD-FISH and subsequent nanoscale secondary ion mass spectrometry (nanoSIMS) analyses [36]. A subsample of the incubated water (50 ml) was fixed and used for CARD-FISH as described above, but without embedding filter sections in agarose. After CARD-FISH and DAPI staining, regions of interest (ROIs) were marked on a laser microdissection microscope (6000 B, Leica) and images of CARD-FISH signals were acquired on an epifluorescence microscope (Axio Imager, Zeiss). After image acquisition, the filters were sputtered with a 7 nm gold layer to create a conductive surface for nanoSIMS analyses. Single-cell ^15^N-uptake from ^15^N-ammonium was determined using a nanoSIMS 50 L (CAMECA), as previously described [37]. Instrument performance was monitored regularly on graphite planchet and on caffeine standards. Due to the small size of AOA (<1 µm), samples were only briefly (10−20 s) pre-sputtered with a Cs^+^ beam (~300 pA) before measurement. Measurements were performed on a field size of 10 × 10 µm to 15 × 15 µm with a dwelling time of 2 ms per pixel and a resolution of 256 × 256 pixels over 25−40 planes. Analysis of the acquired data was performed using the Look@NanoSIMS software package [38]. The potential growth and N-assimilation rates of single cells were calculated as described recently [39]. We did not account for a possible ^15^N-isotope dilution effect introduced by CARD-FISH [40–42], as the strength of the effect depends on the growth phase, activity, and type of cells, which vary strongly in environmental samples. Therefore, the reported potential single cell growth and ammonium assimilation rates are a conservative estimate.

### AOA carbon content calculations

The carbon content of individual AOA cells was inferred according to the nonlinear relationship between carbon mass and cell volume (Eq. 1) as recently established by Khachikyan et al. [43]:

1 $$m_{carbon} = 197 \times V^{0.46},$$

with *m*_*carbon*_ being the mass of carbon in femtograms and *V* being the volume in cubic micrometers of an average AOA cell. *V* was calculated assuming a prolate spheroid cell shape according to Eq. 2 as laid out before [44]:

2 $$V = \frac{\pi }{6} \times W^2 \times L,$$

with *W* being the width and *L* being the length of the cells. AOA cell dimensions were directly obtained from the nanoSIMS measurements, which, unlike fluorescent signals, do not overestimate cell size due to the fluorescence “halo-effect”. The volumetric carbon content of the AOA population was calculated for each time point of our archaeal *amoA* qPCR survey (Fig. 1d) by multiplying the mean carbon content of a single *Nitrosopumilus*-AOA cell (*m*_*carbon*_) with the total abundance of AOA (*A*_*qPCR*_) as inferred by archaeal *amoA* qPCR:

3 $${{{{{{{\mathrm{volumetric}}}}}}}}\;{{{{{{{\mathrm{C}}}}}}}}\;{{{{{{{\mathrm{content}}}}}}}}\left( {{{{{{{{\mathrm{AOA}}}}}}}}} \right) = m_{carbon} \times A_{qPCR}$$

This volumetric C content was integrated over the water column of the hypolimnion and compared to the depth-integrated C content of the phytoplankton, which was inferred from depth-resolved chlorophyll *a* concentrations using the C: Chl *a* (weight:weight) conversion factor of 31.5 [45].

### AOA nitrogen flux calculations

Potential cell-specific ammonia oxidation rates of the AOA population (*R*_*cell-specific*_) were inferred by dividing potential ammonia oxidation rates (*R*_*pot*_) by the total abundance of AOA as inferred by archaeal *amoA* qPCR (*A*_*qPCR*_) obtained at the same time points:

4 $$R_{cell - specific} = \frac{{R_{pot}}}{{A_{qPCR}}}$$

The mean of potential cell-specific ammonia oxidation rates was used to infer the annual volumetric ammonia oxidation rate of the AOA population (*R*_*annual*_) by integrating over our complete data set of total AOA abundances in the years 2017 to 2019:

5 $$R_{annual} = \frac{{\mathop {\sum}\nolimits_1^n {\left[ {\left( {\overline R _{cell - specific}\frac{{A_{qPCR,n} + A_{qPCR,n - 1}}}{2}} \right)\left( {t_n - t_{n - 1}} \right)} \right]} }}{{years}}$$

Whenever average values were used to calculate the mean, e.g., to estimate mean ammonia oxidation rates over the year, the propagation of uncertainty was calculated and the resulting uncertainty provided next to the mean.

## Results & discussion

### AOA account for 8–39% of total archaea and bacteria in the hypolimnion

Depth profiles of total ammonium (NH_4_^+^ + NH_3_) and nitrate showed typical inverse concentration profiles during periods of primary productivity (Fig. 1a–c). Total ammonium decreased and nitrate increased towards the hypolimnion, indicative of active ammonium consumption and nitrate production in hypolimnetic waters, presumably by nitrification (Fig. 1b,c). Hence, we decided to follow annual dynamics of the AOA population in the central part of the hypolimnion at 85 m depth. Copy numbers of Lake Constance-specific archaeal *amoA* genes were determined by quantitative PCR (qPCR). As *amoA* is typically present as a single copy gene in AOA [46], *amoA* copy numbers were compared to total archaeal and bacterial 16S rRNA gene copy numbers to estimate AOA relative abundance. This confirmed the presence of a large prevailing AOA population in the hypolimnion ranging from 8.0 ± 0.9% to 38.9 ± 5.7% of the archaeal and bacterial picoplankton. Absolute abundances ranged between 9.8 × 10^3^ ± 1.0 × 10^4^ and 1.2 × 10^5^ ± 4.9 × 10^4^ *amoA* copies ml^–1^ in winter and summer, respectively, with a mean copy number of 4.3 × 10^4^ *amoA* copies ml^–1^ (Fig. 1d). These qPCR results corresponded well to direct *Nitrososphaeria* cell counts based on catalyzed reporter deposition fluorescence in-situ hybridization (CARD-FISH) at two selected time points (Supplementary Fig. S1). Therefore, we use the terms AOA and *Nitrososphaeria* as synonyms from here on.

### A freshwater-specific AOA species dominates the nitrifier community

**Fig. 2:**
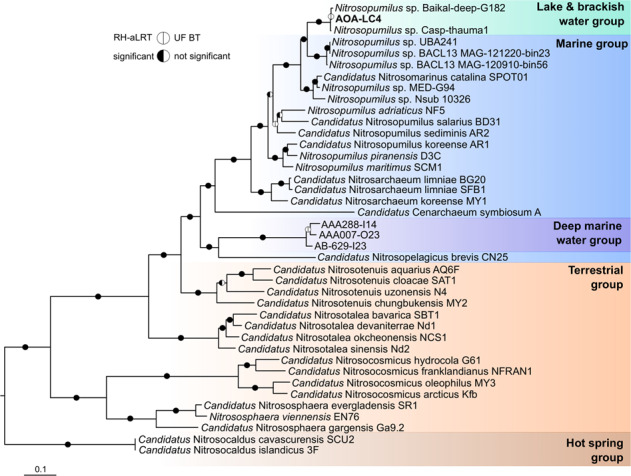
Phylogeny of MAG AOA-LC4 in relation to MAGs retrieved from other major inland water bodies and major lineages within the class *Nitrososphaeria*. The phylogenomic maximum likelihood tree was re-constructed using the IQ-tree algorithm [89] on the basis of a concatenated amino acid alignment of 122 translated single-copy genes that were established by the GTDB-based taxonomy for phylogenetic inference of archaea [25]. Branch support was tested with the Shimodaira–Hasegawa approximate likelihood-ratio test (SH-aLRT; 1000 replicates) and ultrafast bootstrap (1000 replicates) using the IQ-tree software package [89]. Branch support was set as significant at ≥80% for SH-aLRT and ≥95% for ultrafast bootstrap values (black semi-circles for significant and white for non-significant). Non-AOA “Thaumarchaeota” archaeon sp. BS3 (IMG Taxon ID 2721755844), unclassified “Thaumarchaeota” DRTY-7 bin 36 (2263082001), and “Thaumarchaeota” archaeon strain DS1 (2263082000) were used as outgroup. The scale bar indicates 10% estimated amino acid sequence divergence. Accession numbers or respective Taxon IDs can be found in Supplementary Table S3.

To gain insight into the genetic repertoire of the AOA, we conducted a metagenomic survey of hypolimnetic waters covering nine time points from November 2017 to December 2018 (Fig. 1d). Best assembly results were obtained by a co-assembly of the winter datasets November 2017, December 2017 and February 2018, which resulted in a single metagenome-assembled genome (MAG) related to *Nitrososphaeria* that was further refined by long PacBio reads for scaffolding. This resulted in a high quality assembly of eight contigs spanning 1.2 Mbp, with a checkM-estimated [47] coverage of 99% and contamination of 0% (Supplementary Table S1). The 16S rRNA gene of MAG AOA-LC4 was 100% identical to the single 16S rRNA gene-ecotype that was previously shown in an amplicon-based study to outnumber all other AOA, AOB, and comammox populations by at least two orders of magnitude throughout the depth profile of Lake Constance [16] and thus represents the main ammonia oxidizer of this lake. An index of replication (iREP) [48] analysis indicated active replication of the AOA-LC4 population throughout the entire year with a mean of 50 ± 19% and ranging from a minimum of 34% in November/December 2017 to a maximum of 90% in October 2018 (Supplementary Table S1). Although the iRep algorithm was recently questioned to work with population genomes integrating over many co-occurring but heterogeneous close relatives [49], it performs well for uniform population genomes such as pure cultures [48, 50, 51]. We consider the extremely low and highly skewed AOA diversity in Lake Constance [16] to be rather representative of the latter case. The phylogenomic analysis placed AOA-LC4 within a freshwater/brackish water-associated clade of the *Nitrosopumilaceae*, which was distinct from marine representatives (Fig. 2). The closest relatives were MAGs retrieved from Lake Baikal (G182) and from the Caspian Sea (Casp-thauma1), which, together with AOA-LC4, represent a new species within the genus *Nitrosopumilus* based on genome-wide average nucleotide and amino acid identities (Supplementary Fig. S2). Therefore, AOA-LC4 is a representative of AOA in major inland water bodies.

AOA-LC4 encoded the core genetic repertoire of *Nitrosopumilus* species [52, 53]. This included all genes necessary for oxidation of ammonia to hydroxylamine in the canonical arrangement *amoBCxA*, as well as genes with a proposed function in further oxidation of hydroxylamine to nitrite, i.e., genes encoding (putative) multicopper oxidases including a postulated reversely operating nitrite reductase (*“nirK”*) [54, 55]. In addition, AOA-LC4 carries genes for a urea transporter (*dur3*), urea amidohydrolase (*ureABC*), and urease accessory proteins (*ureDEFGH*). Furthermore, it is prototrophic for the vitamins thiamine (B1), riboflavin (B2), biotin (B7), and cobalamin (B12). For carbon fixation, it encodes the energy-efficient variant of the 3-hydroxypropionyl/4-hydroxybutyryl pathway (Supplementary Table S2).

To obtain a complete picture of the nitrifying community, we screened all obtained MAGs and single contigs for the presence of *amoA* as indicator for all ammonia oxidizers, *nxrB* (encoding the beta subunit of nitrite oxidoreductase) as indicator for all nitrite−oxidizing bacteria [56], or both genes in the same MAG/contig as indicator for comammox bacteria [5]. MAG AOB-LC263 encoded the bacterial *amoAB* genes and was affiliated with a novel genus within the *Nitrosomonadaceae* (Proteobacteria) (Supplementary Text and Supplementary Fig. S3, S4). In addition, we found two contigs (AOB-LC199628 and AOB-LC368213), which encoded either *amoAB* or *amoCAB* and were again related to the *Nitrosomonadaceae* (Supplementary Fig. S5). However, both contigs did not group into any of the binned MAGs that fulfilled our completeness criteria (>50%). Two MAGs, NOB-LC32 and NOB-LC29, encoded *nxrAB* and *nxrB*, respectively, and were related to *Nitrospira* lineage II (Nitrospirae) (Supplementary Text and Supplementary Figs. S6−8). Interestingly, we recovered a third *Nitrospira* lineage II MAG, COM-LC224 (Fig. S6), which encoded both *amoA* and *nxrB* (Supplementary Fig. S8, S9). Closer inspection identified the full set of *amoCAB, hao*, and *nxrAB* encoding the ammonia monooxygenase, hydroxylamine reductase, and nitrite oxidoreductase, respectively. This finding was further supported by the affiliation of MAG COM-LC224 to comammox clade B as inferred by independent phylogenetic analysis of its concatenated single copy marker genes as well as its functional marker genes *amoA* and *nxrB* (Supplementary Text and Supplementary Fig. S6, S8, S9). Throughout the year, MAGs AOB-LC263 and COM-LC224 as well as the contigs AOB-LC199628 and AOB-LC368213 were 1–2 orders of magnitude less abundant than AOA-LC4 as inferred from mapping coverage results in the nine metagenomes (Supplementary Table S1). Similarly, MAGs NOB-LC32 and NOB-LC29 were on average one order of magnitude less abundant than AOA-LC4 with the exception of February and October 2018 where they were equally abundant (Supplementary Table S1). This is consistent with results obtained 3 years earlier by 16S rRNA gene amplicon sequencing and *amoA* screening [16].

Based on the low total ammonium concentrations in Lake Constance (on average 0.07 ± 0.04 µM at 85 m depth, Fig. 1) and both the higher substrate affinities (K_m_-apparent) and higher specific substrate affinities (a°) for total ammonium of cultivated *Nitrosopumilus*-AOA compared to AOB, it was not surprising that AOA-LC4 dominated over all detected AOB (although planktonic freshwater AOB have not been characterized for their kinetic properties yet). However, based on the overlapping K_m_-apparent and a°-values of *Nitrosopumilus*-AOA and comammox-*Nitrospira*, one may have expected a more abundant comammox population [57, 58]. A possible explanation could be that comammox are hypothesized to be typical K-strategists being rather adapted to slow growth but higher yield as based on the theory of optimal pathway length [59]. Indeed, the comammox *Nitrospira inopinata* was shown to achieve higher growth yields per mole ammonia at the drawback of lower maximum ammonia oxidation rates as compared to AOAs [57]. These slow growth and metabolic rates of comammox in comparison to the high turnover rate of bacterioplankton biomass in general in the open water column of Lake Constance (1–13 days in the hypolimnion [60]) indicate that comammox-*Nitrospira* were likely outgrown by *Nitrosopumilus*-AOA.

### AOA-LC4 is transcriptionally active throughout the year

**Fig. 3:**
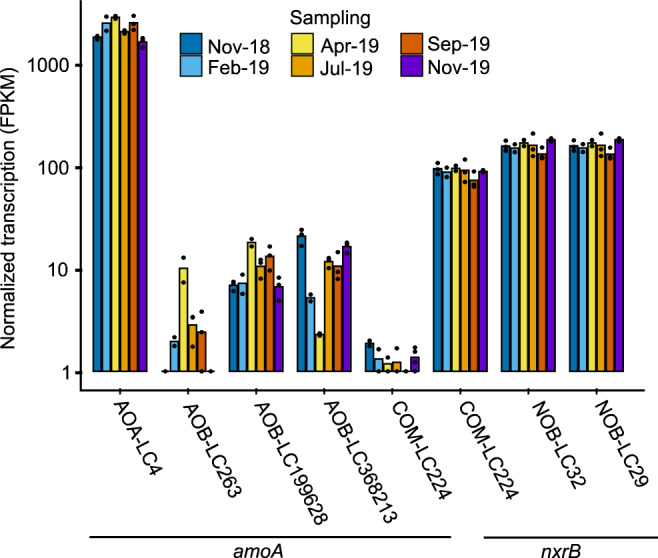
Year-round transcriptional activity of the nitrifying community in the hypolimnion (85 m depth) of Lake Constance based on metatranscriptomics. Transcription of *amoA* (encoding ammonia monooxygenase subunit A) as functional marker for all ammonia oxidizers, *nxrB* (encoding nitrite oxidoreductase subunit B) as functional marker for all nitrite oxidizers, or both genes in the same MAG as indicator for comammox bacteria is shown across all identified nitrifying microorganisms. Bars represent the mean of three replicates, except for the February and April samples, where only two replicates could by analyzed. Individual replicates are indicated by single dots. FPKM values below 1 were set to 1 for a better representation on a log_10_-scale. FPKM, fragments per kilobase of transcripts per million mapped reads.

The dominance of AOA-LC4 was also evident at the transcriptional level. In a metatranscriptome survey covering six time points from November 2018 to November 2019, *amoA* transcription of the AOA-LC4 population was consistently at least two orders of magnitude higher than that of the individual AOB-LC263, AOB-LC199628, AOB-LC368213, or COM-LC224 populations (Fig. 3). Compared to the *nxrB* transcription of the NOB-LC32 and NOB-LC29 populations, the *amoA* transcription of AOA-LC4 was consistently one order of magnitude higher, reflecting the relative abundance estimates based on normalized metagenomic coverage (Supplementary Table S1) and 16S rRNA gene amplicons [16]. Interestingly, the transcription of the COM-LC224 *amoA* gene was one order of magnitude lower than of its *nxrB*, suggesting that nitrite oxidation may predominate over complete ammonia oxidation to nitrate in this microorganism, potentially driven by a shift from a K- to an r-strategy as outlined above. Alternatively, the represented two enzymes have different turnover times or regulation mechanisms.

**Fig. 4:**
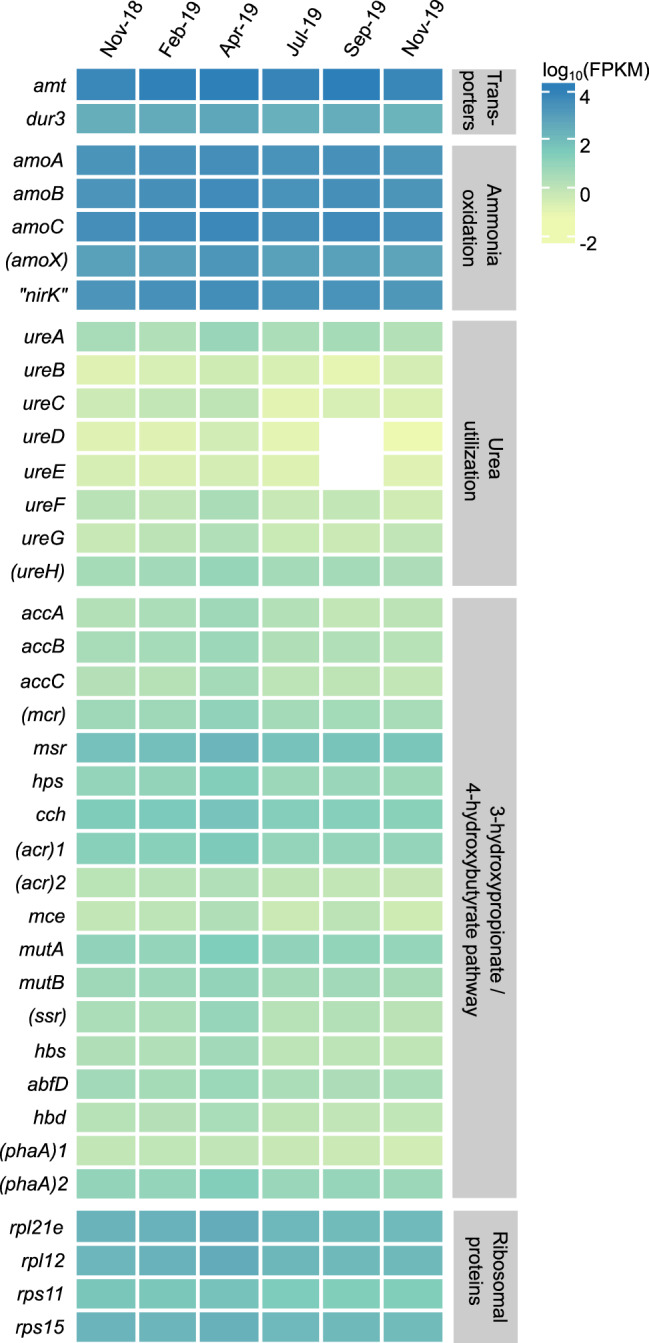
Seasonally resolved transcription of genes involved in the nitrogen and carbon metabolism of AOA-LC4 at 85 m depth. As reference housekeeping genes, the two highest transcribed genes encoding either proteins of the large (*rpl12, rpl21e*) or small ribosomal subunit (*rps11*, *rps15*) are shown at the bottom. Transcription values are represented as log_10_-transformed mean FPKM (fragments per kilobase of transcripts per million mapped reads) values for every time point. Values represent the mean of three replicates, except for the February and April samples, where only two replicates could by analyzed. No transcription of a specific gene is indicated in white. Putatively annotated genes are given in brackets; gene duplicates were consecutively numbered.

Detailed analysis of the seasonally resolved AOA-LC4 population transcriptome identified *amoABC* among the top ten transcribed genes at a steady level with little variation throughout the yearly cycle (Fig. 4, Supplementary Table S2). Transcription of *amoABC* was highly correlated (*r*_*S*_ ≥ 0.8) to each other and to transcription of “*nirK”* and a gene encoding a membrane-anchored PEFG-CTERM domain-containing multicopper oxidase (locus JW390_30004) (Supplementary Fig. S10). The latter two were previously postulated to be involved in the yet unresolved archaeal pathway of ammonia oxidation to nitrite [53–55, 61]. On average, *amoABC* showed a 16 to 119 fold higher transcription than the highest transcribed genes encoding either proteins of the large (*rpl12*, *rpl21e*) or small ribosomal subunit (*rps11*, *rps15*), which we used as reference housekeeping genes (Fig. 4). Also *amt* and *cdvB*, encoding a high-affinity ammonium transporter and the putative cell division protein B2, respectively, were consistently found among the top ten highest transcribed genes. Since the active center of the archaeal ammonia monooxygenase is postulated to face the outside of the cytoplasmic membrane [55], the high transcriptional levels of *amt* imply that ammonia was transported into the cell for ammonium assimilation, most likely for further biomass production as evidenced by the high transcriptional levels of *cdvB*. This is in line with the consistent transcription of genes encoding the 3-hydroxypropionate/4-hydroxybutyrate pathway for CO_2_ fixation, however, at 1 to 300 fold lower transcriptional levels as compared to the selected ribosomal reference genes (Fig. 4, Supplementary Table S2). Together, this resembles (meta-)transcriptomic data of actively growing AOA species in culture and in open marine waters with AOA-driven ammonia oxidation activity [54]. Therefore, we conclude that the AOA-LC4 population was actively oxidizing ammonia throughout the year. In contrast, genes associated with urea utilization were much less transcribed. While *dur3* (encoding an urea transporter) was transcribed similar to the four highest transcribed ribosomal protein genes, *ureABC* (encoding urease) showed a 12 to 988 fold lower transcription. The same was true for *ureDEFGH* (encoding [putative] urease accessory proteins) with transcriptional levels either below the detection limit, or 43 to 1507 fold lower. However, low transcription of urease and urease-associated genes has previously been shown to be a poor predictor for lack of urea-based ammonia oxidation of AOA [54].

### AOA predominate ammonium assimilation in the hypolimnion

**Fig. 5:**
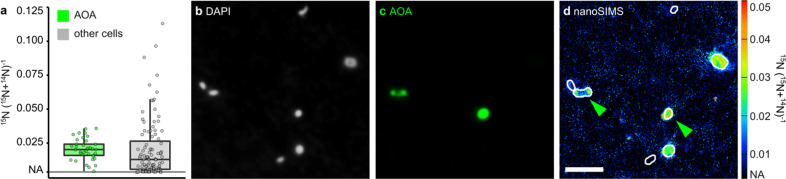
Single-cell ammonium uptake by AOA and non-target picoplankton cells in hypolimnetic water from 85 m depth determined by nanoSIMS combined with CARD-FISH analysis. (**a**) ^15^N- enrichment of AOA (*n* = 37) and non-target cells (*n* = 105) after addition of ^15^N-ammonium. Representative images of (**b**) DAPI-stained cells, (**c**) corresponding CARD-FISH signals with a *Nitrososphaeria*-specific probe, and (**d**) the corresponding ^15^N-enrichment determined by nanoSIMS; green arrows indicate AOA; NA Natural abundance; scale bar = 2 µm.

To link transcriptional to metabolic activity, we performed short-term (48 h) ^15^NH_4_^+^-labeling experiments in parallel to our metatranscriptomic survey in November 2019. As described above, transcriptional activity of the AOA-LC4 population in these samples was high and exceeded other ammonia oxidizers’ transcriptional levels (Fig. 3). As a measure of metabolic activity, incorporation of ^15^N-ammonium was analyzed at the single-cell level using nanoscale secondary ion mass spectrometry (nanoSIMS) coupled to CARD-FISH identification of AOA cells. All measured AOA cells (*n*_cells_ = 37) were metabolically active and incorporated ammonium at an average potential rate of 5.04 ± 3.01 amol NH_4_^+^ cell^–1^ d^–1^. The AOA population was significantly more enriched in ^15^N than the remaining picoplankton community (*n*_cells_ = 105, non-parametric Mann–Whitney *U*-test, *p* < 0.005) (Fig. 5). This was in line with a cross-comparison of transcript levels of ammonia transporters detected across all metagenomes. Also here, 95–98% of all transcripts (mean 97%) were attributed to *amt* of AOA-LC4. In addition, AOA-LC4 dominated the transcript levels of all detected urea transporter genes (mean 74%, range 61–83%) (Supplementary Table S2).

Using the nanoSIMS results, we calculated potential single-cell N-based growth rates of 0.012 ± 0.006 d^–1^ for AOA at the in-situ temperature of 4°C. These rates are one order of magnitude lower than those previously observed in pure cultures of *Nitrosopumilus maritimus* [3] or for AOA in marine subtropical waters [31]. This can be explained by the higher incubation temperature of 28°C in the latter two studies. It should, however, be noted that two other studies in cold marine waters (ice-water to 4°C) [62, 63] reported AOA growth rates comparable to those of *N. maritimus* at 28°C [3]. The nanoSIMS measurements were also used to quantify single AOA cell dimensions. The average AOA cell in Lake Constance is around 0.54 ± 0.11 µm long and 0.39 ± 0.10 µm wide and has a volume of 0.048 ± 0.035 µm³. Several methods have been used in the literature to estimate cellular C-content [63–66]. Here, we use the empirical relationship between cell volume and C-content established by Khachikyan et al. [43] across different microbial species, which directly determined cell mass on the single cell level [67]. Using this established empirical relationship between cell volume and cellular C-content, the average Lake Constance AOA cell has a C-content of 46 ± 15 fg-C cell^−1^. This cellular C-content is higher than that of marine and cultivated *Nitrosopumilus* strains (8–17 fg-C cell^−1^ [63–66]. However, this is not unexpected given the 4.4-fold larger volume of Lake Constance AOA compared to, e.g., *N. maritimus* [43]. Integration of the inferred cellular C-content over the total abundance of Lake Constance AOA (Fig. 1d) results in 0.5–5.5 mg-C m^‒3^ (mean 2.0 mg-C m^‒3^) stored in AOA cells in the hypolimnion. Compared to phytoplankton biomass, which we inferred from depth-integrated chlorophyll *a* measurements, and considering that the hypolimnion extends over 120 m at our sampling station, this AOA-stored carbon makes up 3.3–30.6% (average 12.3%) of carbon stored in phytoplankton over the year´s cycle (Fig. 1e).

### AOA oxidize up to 1763 metric tons of ammonium-N per year in Lake Constance

All independent lines of evidence obtained in this study arrived at the conclusion that the AOA-LC4 population is driving ammonia oxidation in Lake Constance, with a negligible contribution by AOB and comammox bacteria. This natural setting posed the unique opportunity to quantify the ecosystem function exerted by planktonic AOA and to link this information to a single ecotype. We followed potential ammonia oxidation rates in the hypolimnion (85 m depth) by ^15^NH_4_^+^ incubations over four independent time points from June to November 2019. Potential ammonia oxidation rates were very similar within this time period with an average of 6.0 ± 0.9 nmol l^‒1^ d^‒1^ (Table 1). Although the obtained rates are potential rates, the absence of an obvious activity lag phase and the linearity of the activities (*R*^2^ = 0.91–1.00) throughout the incubation time (48 h) point towards an active AOA-LC4 population in situ (Supplementary Fig. S11). The elevated total ammonia concentrations (10 µM) used to measure ammonia oxidation rates are in a range in which the maximum reaction rate of *Nitrosopumilus* species can be expected [58]. Our previously published estimate of the nitrification rate in Lake Constance was considerably higher but was based on ^15^N-dilution from the nitrate pool [16] instead of measuring direct conversion of ^15^N-ammonium to ^15^N-nitrite/nitrate (this study). In addition, the previous single time point estimate was based on a high variation of residual ^15^N across replicates. Therefore, we consider the replicated potential rate measurements presented in this study to be more reliable. In addition, comparison to rates obtained from other oligotrophic and deep lakes such as Lake Superior, USA [19, 68], or Lake Taupo, New Zealand [69], showed that the obtained rates in Lake Constance were in the same range. The same was true when our data were compared to oligotrophic marine environments dominated by AOA, where similar rates were observed in oligotrophic regions of the Atlantic Ocean [70, 71] and one order of magnitude lower rates in the oligotrophic equatorial Pacific Ocean [72, 73]. In contrast, volume-based ammonia oxidation rates in considerably smaller lakes with higher trophic status and higher total ammonium availabilities can be 1–5 orders of magnitude greater than those observed in large oligotrophic water bodies such as the hypolimnion of Lake Constance [74–77].

**Table 1:** Potential ammonia oxidation rates (*n* = 3) and derived potential cell-specific AOA-driven ammonia oxidation rates in the hypolimnion of Lake Constance.

Sampling date (yyyy-mm-dd)	Potential ammonia oxidation rate in the hypolimnion (nmol l^–1^ d^–1^)	*archaeal amoA* copy numbers (10^4^ copies ml^–1^)	Potential cell-specific ammonia oxidation rate (fmol cell^–1^ d^–1^)
2019-06-18	5.67 ± 0.38	2.50 ± 0.63	0.23 ± 0.06
2019-07-29	4.61 ± 0.39	n.d.	‒
2019-08-28	6.06 ± 0.37	n.d.	‒
2019-11-05	7.72 ± 0.66	3.81 ± 1.55	0.20 ± 0.08

n.d. Not determined

Cell-specific ammonia oxidation rates were inferred from bulk ammonia oxidation rates divided by the respective absolute abundance of AOA measured by archaeal *amoA* qPCR.

Measured potential rates in Lake Constance were normalized by archaeal *amoA* copy numbers for the June and November samples to determine potential cell-specific ammonia oxidation rates of the AOA population. At both time points, potential cell-specific rates were highly similar and averaged to 0.21 ± 0.11 fmol cell^–1^ d^–1^ (Table 1). Like the calculated AOA growth rates, these cell-specific ammonia oxidation rates at 4°C are one order of magnitude lower than those observed for cultivated marine *Nitrosopumilus* species at 22 to 28°C [3, 78], or for AOA in marine subtropical waters [39], as would be expected from the Q_10_ temperature coefficient rule [79]. We used the obtained potential cell-specific rates to infer the upper limit of AOA-driven nitrification over our complete data set of AOA abundances in Lake Constance (Fig. 1d). As a result, we estimate that about 46 mg NH_4_^+^ m^–3^ y^–1^ can be oxidized by the AOA population. This translates to a total of 1.76 × 10^9^ g (1763 metric tons) N-NH_4_^+^ that can be oxidized per year if integrated over the whole lake´s hypolimnion (38.1 km^3^). Since we cannot rule out local maxima of ammonia oxidation along the water column, e.g., due to temperature and total ammonium gradients during summer stratification, and given that we obtained the rates from water sampled at the center of the hypolimnion and at ca. 4°C, the maximum capacity for AOA-driven ammonia oxidation might be even higher. In comparison, 15.7 × 10^9^ g of biomass-N are annually produced by primary production in the photic zone of Lake Constance, when considering the annual primary productivity rate of 220 g C m^–2^ y^–**1**^ [80], 473 km^2^ surface area of Lake Constance [24] and the Redfield ratio (C:N = 106:16). This results in potential AOA-converted N corresponding to 11% of photosynthetic biomass-N produced in the photic zone of Lake Constance. AOA-driven ammonia oxidation results eventually in the formation of nitrate. In addition, 13.7 × 10^9^ g dissolved N (mainly in the form of nitrate) enter Lake Constance annually via riverine input, treated outflow of wastewater treatment plants, rain, and diffusive transport [81]. As such, nitrification contributes an upper limit of roughly 4% of the nitrate entering the nitrate pool annually (42.8 × 10^9^ g N-nitrate) of Lake Constance [24].

### Epilog proposal of “Candidatus Nitrosopumilus limneticus”

Based on its phylogenetic placement, gANI values and habitat preference, we propose a new species name for AOA-LC4: “*Candidatus* Nitrosopumilus limneticus sp. nov.” (lim.ne’ti.cus. N.L. masc. adj. *limneticus* (from Gr. *limne*, lake, swamp) belonging to a lake). “*Candidatus* Nitrosopumilus limneticus” encodes and transcribes genes essential for dissimilatory ammonia oxidation to nitrite and autotrophic CO_2_ fixation via the 3-hydroxypropionyl/4-hydroxybutyryl pathway. Its preferred habitat is the oxygenated hypolimnion of oligotrophic freshwater lakes.

## Conclusion

Over the last two decades, reports have accumulated that deep oligotrophic lakes sustain large populations of *Nitrososphaeria* (“Thaumarchaeota”) in their hypolimnion [10, 15–20]. Our study links these large archaeal populations to ammonia oxidation activity and quantifies this important ecosystem service at the single-cell, population, and ecosystem levels. We provide compelling evidence that AOA in a deep oligotrophic lake play an equally important role in the nitrogen cycle—both in terms of their relative abundance and nitrogen fluxes—as their counterparts in marine ecosystems [21, 82]. Lakes are considered sentinels of climate change responding to rising annual temperatures by changes in their physical, chemical, and biological properties [83]. Lake Constance is no exception; its surface waters have become increasingly much warmer, with an annual average increase of 0.9°C observed between 1962 to 2014 [84]. Warming is predicted to continue at 0.03°C y^−1^ resulting in increasing thermal stratification and concomitant increased deoxygenation of deep hypolimnetic waters [84]. Here, we show that ammonia oxidation is a key process in the lake’s nitrogen cycle driven by a single freshwater AOA ecotype, designated as “*Candidatus* Nitrosopumilus limneticus”. The results support the general view that alpha diversity of AOA in freshwater lakes is extremely low [85–88]. This raises the question how resilient this ecosystem service is to changes in the physical and chemical properties of freshwater lakes in the face of climate change. Since nitrification prevents the accumulation of harmful ammonium, answers to this question are interlinked with our quest for drinking water supply and the quality of freshwater bodies for fisheries and other lacustrine fauna.

## Supplementary information

Supplementary Information

Supplementary Table 1

Supplementary Table 2

Supplementary Table 3

## Data Availability

All metagenome and metatranscriptome sequences as well as annotated MAGs are available at NCBI under bioproject number PRJNA691101. AOA abundance data (qPCR), total ammonium, nitrate and chlorophyll *a* concentrations, temperature and oxygen measurements and ammonia oxidation rates for the time span of our study are deposited at PANGAEA (10.1594/PANGAEA.934577).
