# Supplementary material for: Quantification of archaea-driven freshwater nitrification from single cell to ecosystem levels
Source: ISME J. 2022 Mar 8;16(6):1647–56. doi: 10.1038/s41396-022-01216-9 (PMC9122916; doi:10.1038/s41396-022-01216-9)
Supplement: Supplementary file 1 — Supplementary Information [file 41396_2022_1216_MOESM1_ESM.pdf]

Supplementary information for

Quantification of archaea-driven freshwater nitrification from single cell to ecosystem levels

Franziska Klotz[1], Katharina Kitzinger[2], David Kamanda Ngugi[3], Petra Büsing[3], Sten Littmann[2], Marcel M. M. Kuypers[2], Bernhard Schink[1], and Michael Pester[1,3,4]

1. *Department of Biology, University of Konstanz, Universitätsstrasse 10, Konstanz, D-78457, Germany*
2. *Max Planck Institute for Marine Microbiology, Celsiusstrasse 1, D-28359 Bremen, Germany.*
3. *Leibniz Institute DSMZ – German Collection of Microorganisms and Cell Cultures, Inhoffenstr. 7B, D-38124 Braunschweig, Germany*
4. *Technical University of Braunschweig, Institute for Microbiology, Spielmannstrasse 7, D-38106 Braunschweig, Germany*

Supplementary Methods

Measurements of environmental parameters

Vertical profiles of temperature and oxygen were measured down to the lake sediment with a multi-sampling probe (RBR Ltd.; Ottawa, Canada, Sea & Sun Technology GmbH, Trappenkamp, Germany or bbe Moldaenke GmbH, Schwentinental, Germany) at a resolution of 0.5 m and on the following dates (yyyy-mm-dd): 2017-11-08, 2017-11-21, 2017-12-05, 2018-01-09, 2018-01-23, 2018-02-06, 2018-02-20, 2018-03-13, 2018-03-27, 2018-04-10, 2018-04-24, 2018-05-22, 2018-06-05, 2018-06-19, 2018-07-03, 2018-07-31, 2018-08-14, 2018-09-21, 2018-10-09, 2018-10-23, 2018-11-22, 2018-12-04, 2018-12-18, 2019-01-11, 2019-02-12, 2019-02-26, 2019-03-12, 2019-03-27, 2019-04-09, 2019-04-25, 2019-06-18, 2019-07-02, 2019-07-31, 2019-08-13, 2019-08-28, 2019-09-24, 2019-10-08, 2019-10-23, 2019-11-04, 2019-11-19.

Vertical profiles of nitrate and total ammonium (NH_4^+ + NH_3) were measured for the same dates as used for qPCR analyses. Nitrate and total ammonium concentrations were determined using the auto-analyzer and Seal analytics methods G-172-96 Rev. 12 and G-171-96 Rev. 14, respectively (SEAL Analytical GmbH, Norderstedt, Germany). For these measurements, 20 ml water each from 13 depths at 1, 5, 10, 15, 20, 25, 30, 40, 50, 60, 85, 110 and 135 m were taken, filtered through a Chromafil® GF/PET-20/25 filter (pore size 1.0 and 0.2 µm, VWR, Vienna, Austria) and stored at –20°C until analysis. Samples obtained between July and November 2019 were measured by an alternative method: Nitrate was measured by ion chromatography (S150 Chromatography System, SYKAM) and

total ammonium was measured fluorometrically by the *ortho*-phthaldialdehyde method [1]. Chlorophyll *a* was sampled from 22 depths over a gradient of 0−60 m and analyzed spectrophotometrically after extraction in hot ethanol as described previously [2], but without correcting for pheopigments.

DNA and RNA extraction

For DNA extraction, 0.22 µm-filters (47 mm diameter) were placed in 2 ml-screw cap tubes and vortexed for 15 min in a solution containing 500 µl TE-buffer (10 mM Tris-HCl pH 8.0, 1 mM EDTA), 12.5 µl 20% sodium lauryl sulfate-solution (SLS; Sigma-Aldrich, Taufkirchen, Germany), 500 µl phenol-chloroform-isoamylalcohol 25:24:1 (Carl Roth GmbH), and 200 µl pre-combusted zirconium beads (0.1 mm in diameter, Carl Roth GmbH, Karlsruhe, Germany). After centrifugation (4°C, 10 min, 18,620 ×*g*), the supernatant was washed once with 500 µl chloroform-isoamylalcohol 24:1 (Carl Roth GmbH). This mixture was centrifuged again and DNA precipitated from the separated supernatant over night at −20°C using a mixture of 0.1 volume 3 M sodium acetate (pH 4.8), 2.5 volume molecular grade ethanol (Carl Roth GmbH) and 1 µl glycogen (20 mg ml^{-1}, Thermo Fisher Scientific, Waltham, MA, USA). Precipitated DNA was centrifuged, washed twice with 80% molecular grade ethanol, and dissolved in nuclease-free water (MP biomedicals, Eschwege, Germany). RNA was removed by an RNase ONE™ ribonuclease treatment following kit instructions (Promega, Fitchburg, WI, USA) and DNA samples were stored at –20°C until processing.

For RNA extraction, 0.22 µm-filters (142 mm diameter) filters were cut with a sterilized scissor into thirds and extracted as described above except for the following modifications: filters were extracted in extraction buffer (50 mM sodium acetate and 10 mM EDTA, pH 4.2) with 0.025% SLS (Sigma-Aldrich) and phenol-chloroform-isoamylalcohol 25:24:1 (Roti-Aqua-P/C/I 4.5-5.0, Carl Roth GmbH). Washing of the aqueous phase with chloroform-isoamylalcohol 24:1 was done in the presence of 0.1 volume 3 M sodium acetate. RNA was finally precipitated with 1 volume ice-cold isopropanol in the presence of 1 µl glycogen (35 mg ml^{-1}, RNase-free, VWR), washed as stated above, and eluted in nuclease-free water (MP biomedicals). DNA was digested with the TURBO DNA-free™ kit (Thermo Fisher Scientific) and RNA samples were stored afterwards at –80°C until sequencing.

Determination of AOA abundance by CARD-FISH

Before CARD-FISH, cells on the filter sections were immobilized by embedding in 0.1% low-gelling agarose (Metaphor). CARD-FISH was performed using a specific HRP-labeled oligonucleotide probe for *Nitrososphaeria* (HRP-labeled Thaum726 [GCTTTCATCCCTCACCGTC] and unlabeled competitors [Thaum726_compA: GCTTTCGTCCCTCACCGTC, Thaum726_compB: GCTTTCATCCCTCACTGTC]) [3, 4]

as described previously [5]. Negative controls using NonEUB [6] to exclude unspecific signals were performed according to a defined protocol [7]. Briefly, endogenous peroxidases were inactivated by incubation in 0.01 M HCl for 10 min. Cells were permeabilized by HCl (0.1 M HCl for 1 min) and subsequently washed with MilliQ water. Filter pieces were hybridized with HRP probes and the respective competitor probes at 25% formamide concentration at 46°C for up to 3 h. After a 5 min washing step at 48°C and HRP probe equilibration in 1× PBS for 5 to 15 min, signal amplification was performed with OregonGreen488-labeled tyramides at 48°C for 30 min. Cells were counterstained with 4',6-diamidino-2-phenylindole (DAPI, 10 µg ml^{-1}, 5 min at room temperature). Filter sections were mounted onto glass slides, and embedded in a 4:1 mixture of Citifluor AF1 and Vectashield (Citifluor Ltd, London, UK; Vector Laboratories, Burlingame, CA, USA). *Nitrososphaeria* and DAPI signals were counted on an Axiophot or Axioplan 2 microscope (Zeiss, Germany).

Next generation sequencing and bioinformatics processing

Metagenome sequencing libraries were prepared with the NEBNext® Ultra™ DNA Library Prep Kit for Illumina® (New England Biolabs GmbH, Frankfurt am Main, Germany) and sequenced on an Illumina NextSeq500 sequencer using 2 × 150 bp. This resulted in $0.6–2.7 \times 10^8$ reads per metagenome with an average of 1.3×10^8 reads (8.8–41 Gbp, average 20 Gbp). Raw Illumina reads were quality checked with FastQC v.0.11.8 [8] and subsequently quality filtered and trimmed using Sickle v1.33 [9]. Thereafter, reads were assembled with Megahit v1.1.2 [10] and binned with maxbin2 v2.2.4 [11]. A co-assembly of the November 2017, December 2017, and February 2018 metagenomes resulted in the best AOA bin. To refine this bin, DNA from November 2017 was sequenced in addition by PacBio sequencing on a Sequel instrument (Pacific Biosciences, Menlo Park, CA, USA) using circular consensus sequencing with a target length of 2 kbp. Raw PacBio reads were quality controlled with smrt analysis using an accuracy of 0.999; the number of resulting CCS bases was 0.55 Gbp. The original Illumina AOA bin was used as trusted contigs in spades v3.11.1 [12] and re-assembled with Sequel-reads for gap closure. A subsequent binning in metabat2 v2.12.1 [13] resulted in the final MAG AOA-LC4. MAGs related to AOB, NOB or comammox could not be further refined by long PacBio reads. All MAGs were tested for completeness, strain heterogeneity, and contamination using CheckM v1.0.7 [14] and for their index of replication using iRep v1.10 [15]. MAGs and single contigs were screened for the presence of the functional marker genes *amoA* and *nxrB* by both blastp v2.10.1 [16] (e-value threshold 1^{-10}) and hmm-search v3.3 [17] (e-value threshold 1^{-5}). The latter was based on hmm-models retrieved from the fungene database with manual curation [amoA_AOA.hmm (Feifei Liu), amoA_AOB.hmm (RDP) amoA_comammox.hmm (Yang Ouyang), nxrB.hmm (RDP), fungene.cme.msu.edu] [18]. MAG AOA-LC4 was annotated using the Microscope platform [19]. The automated annotation was manually refined using annotation rules laid out before [20]. Additional

MAGs were annotated with PROKKA v1.12 [21] and curated manually for their functional marker genes *amoABC, hao,* and *nxrAB,* where appropriate.

For metatranscriptome sequencing, messenger RNA (mRNA) was enriched from total RNA extracts by depleting ribosomal RNA with the Ribo-off rRNA Depletion Kit for bacteria (Vazyme, Nanjing, China). Thereafter, the sequencing library was prepared with the TruSeq® Stranded mRNA Library Prep (Illumina) and sequenced on a NextSeq500 sequencer using 2 × 150 bp. The sequencing depth ranged between 0.7−1.9 × 10^8 reads per metatranscriptome with an average of 1.2 × 10^8 reads (10−27 Gbp, average 17 Gbp). Raw reads were quality filtered and trimmed using trimmomatic v0.38 [22] and the fastx toolkit v0.0.14 (hannonlab.cshl.edu/fastx_toolkit). Residual ribosomal reads were removed using SortMeRNA v2.1b [23]. Curated metatranscriptome reads were mapped against MAGs and contigs of interest using bowtie2 v2.30 [24] to determine the transcription levels of individual genes. Subsequent network analysis of co-transcribed genes of MAG AOA-LC4 was based on genes with transcription values higher than the median transcription of all AOA-LC4 genes (35.35 FPKM). Transcription values were correlated pairwise using Spearman correlation; only significant (FDR-corrected p-value < 0.05) correlations with a correlation coefficient of $r_S > 0.8$ were further processed. For the final network construction, only genes, which correlated in their transcriptional response to at least two of the either *amoA*, *amoB* or *amoC* were taken into account. The network was created with the R package igraph v1.2.5 [25] and refined with cytoscape v3.8.1 [26].

A gene-centric analysis was performed to gain an overview of all ammonia transporter (*amt*) and urea transporter (*dur3)* or urea transport system substrate-binding protein (*urtA*) gene sequences in Lake Constance. Therefore, single assemblies of all 9 metagenomes were annotated with DRAM [27]. The retrieved *amt*, *dur3* and *urtA* sequences were de-replicated with cd-hit-est [28, 29]. Curated metatranscriptomes were mapped onto unique *amt*, *dur3* and *urtA* sequences using bowtie2 v2.30 [24] to compare their transcription levels with AOA-LC4.

Phylogenetic analyses

Phylogenomic analyses of the nitrifying MAGs were performed on the basis of concatenated amino acid alignments of 122 translated archaeal or 120 bacterial single copy genes [30, 31]. Alignments were generated using GTDB-Tk v0.3.2 [32] and maximum likelihood trees were constructed using IQ-tree v1.6.12 [33]. Branch support was tested with the Shimodaira–Hasegawa approximate likelihood-ratio test [34] and ultrafast bootstrap [33] options in IQ-tree. Genome-wide average nucleic and amino acid identities (ANI and AAI, respectively) were calculated with the online tool (enve-omics.ce.gatech.edu) developed previously [35] using default settings. Maximum likelihood trees for AOB-*amoA*, comammox-*amoA* and NOB/comammox-*nxrB* genes were constructed in IQ-tree based on manually curated alignments established in ARB v6.0.3 [36].

Supplementary Results

Phylogenetic analysis of bacterial ammonia oxidizers

Phylogenomic maximum likelihood tree construction revealed that MAG AOB-LC263 formed a stable cluster with other freshwater MAGs, which represented a sister clade to *bona fide Nitrosospira* species (Fig. S3). This was corroborated by phylogenetic analysis of its *amoA* gene (Fig. S5). Closest relatives of AOB-LC263 were MAGs retrieved from Lake Baikal, the Great Lakes, and Lake Biwa. Based on the currently proposed species and genus delineation thresholds of ca. <95% ANI and <65% AAI, respectively [37], AOB-LC263 would represent a new species and genus within the *Nitrosomonadaceae* (Fig. S4). The phylogenetic affiliation of contigs AOB-LC199628 and AOB-LC368213 could only be assessed based on their *amoA* genes. Contig AOB-LC199628 clustered in a stable clade consisting of environmental sequences that was distinct from the AOB-LC263 and *Nitrosospira* clusters. Its closest cultured relative was *Nitrosospira* sp. Np39-19 as based on 78.3% *amoA* nucleotide identity. Contig AOB-LC368213 clustered within sequences affiliated with *Nitrosomonas* species with its closest cultured relative being *Nitrosomonas ureae* Nm10 with 89.9% *amoA* nucleotide identity (Fig. S5). Comparison of the retrieved *amoA* sequences to a previous bacterial *amoA* clone library obtained from Lake Constance waters [38] revealed that the *amoA* gene of contig AOB-LC199628 was 100% identical (nucleic acid identity) to clone BmcYyy23.2 (MH780622.1) from OTU1 [38]. Furthermore, the *amoA* of MAG AOB-LC263 was 99.8% identical to clone BmcYyy33 (MH780602.1) from OTU2. Since only two OTUs were detected previously [38], contig AOB-LC368213 had no representatives in the earlier *amoA* clone library.

Phylogenetic analysis of nitrite-oxidizing bacteria and comammox bacteria

Phylogenomic maximum likelihood tree construction placed the two MAGs NOB-LC29 and NOB-LC32 into *Nitrospira* lineage II but outside the intra-lineage comammox clades A and B (Fig. S6). This was corroborated by phylogenetic analysis of their *nxrB* genes (Fig. S8). The two MAGs shared an ANI and AAI of 85% and 85%, respectively, indicating that they represent two separate species within the genus *Nitrospira* (Fig. S7). Phylogenomic tree construction of MAG COM-LC224 placed it into comammox clade B within *Nitrospira* lineage II (Fig. S6), which was corroborated by phylogenetic placement of its single genes *nxrB* and *amoA* (Fig. S8 and S9). Interestingly, COM-LC224 showed <65% AAI to both type and *Candidatus* species within the genus *Nitrospira* (Fig. S7), but at the same time exhibited AAI values of >65% with freshwater *Nitrospira* like NOB-LC29 and NOB–LC32. Without further data, its affiliation at the taxonomic rank of a genus is currently inconclusive.

References

1. Holmes RM, Aminot A, Kérouel R, Hooker BA, Peterson BJ. A simple and precise method for measuring ammonium in marine and freshwater ecosystems. Can J Fish Aquat Sci

1999;56:1801–1808.

2. Tilzer MM. The importance of fractional light absorption by photosynthetic pigments for phytoplankton productivity in Lake Constance. Limnol Oceanogr 1983;28:833–846.

3. Beam JP. Geobiological interactions of archaeal populations in acidic and alkaline geothermal springs of Yellowstone National Park, WY, USA. 2015. Montana State University.

4. Sauder LA, Albertsen M, Engel K, Schwarz J, Nielsen PH, Wagner M, et al. Cultivation and characterization of *Candidatus* Nitrosocosmicus exaquare, an ammonia-oxidizing archaeon from a municipal wastewater treatment system. ISME J 2017;11:1142–1157.

5. Kitzinger K, Padilla CC, Marchant HK, Hach PF, Herbold CW, Kidane AT, et al. Cyanate and urea are substrates for nitrification by Thaumarchaeota in the marine environment. Nat Microbiol 2019;4:234–243.

6. Wallner G, Amann R, Beisker W. Optimizing fluorescent in situ hybridization with rRNA-targeted oligonucleotide probes for flow cytometric identification of microorganisms. Cytometry 1993;14:136–143.

7. Pernthaler A, Pernthaler J, Amann R. Sensitive multi-color fluorescence in situ hybridization for the identification of environmental microorganisms. In: Kowalchuk G, de Bruijn FJ, Head IM, Akkermans ADL, van Elsas JD (eds). Molecular Microbial Ecology Manual, 2nd ed. 2004. Kluwer Academic Publishers, Dordrecht, Boston, London, pp 711–726.

8. Andrews S. FASTQC: A quality control tool for high throughput sequence data. https://www.bioinformatics.babraham.ac.uk/projects/fastqc/. [Software]. 2010.

9. Joshi N, Fass J. Sickle: A sliding-window, adaptive, quality-based trimming tool for FastQ files (Version 1.33) [Software]. 2011.

10. Li D, Liu CM, Luo R, Sadakane K, Lam TW. MEGAHIT: An ultra-fast single-node solution for large and complex metagenomics assembly via succinct de Bruijn graph. Bioinformatics 2015;31:1674–1676.

11. Wu YW, Simmons BA, Singer SW. MaxBin 2.0: An automated binning algorithm to recover genomes from multiple metagenomic datasets. Bioinformatics 2016;32:605–607.

12. Nurk S, Meleshko D, Korobeynikov A, Pevzner PA. metaSPAdes: a new versatile metagenomic assembler. Genome Res 2017;27:824–834.

13. Kang DD, Li F, Kirton E, Thomas A, Egan R, An H, et al. MetaBAT 2: an adaptive binning algorithm for robust and efficient genome reconstruction from metagenome assemblies. PeerJ 2019;7:e7359.

14. Parks DH, Imelfort M, Skennerton CT, Hugenholtz P, Tyson GW. CheckM: assessing the quality of microbial genomes recovered from isolates, single cells, and metagenomes. Genome Res 2015;25:1043–1055.

15. Brown CT, Olm MR, Thomas BC, Banfield JF. Measurement of bacterial replication rates in microbial communities. Nat Biotechnol 2016;34:1256–1263.

16. Altschul SF, Gish W, Miller W, Myers EW, Lipman DJ. Basic local alignment search tool. J Mol Biol 1990;215:403–410.

17. Eddy SR. A new generation of homology search tools based on probabilistic inference. Genome Inform 2009;23:205–211.

18. Fish JA, Chai B, Wang Q, Sun Y, Brown CT, Tiedje JM, et al. FunGene: the functional gene pipeline and repository. Front Microbiol 2013;4:291.

19. Vallenet D, Calteau A, Cruveiller S, Gachet M, Lajus A, Josso A, et al. MicroScope in 2017: an expanding and evolving integrated resource for community expertise of microbial genomes. Nucleic Acids Res 2016;45:D517–D528.

20. Hausmann B, Pelikan C, Rattei T, Loy A, Pester M. Long-term transcriptional activity at zero growth of a cosmopolitan rare biosphere member. mBio 2019;10:e02189-18.

21. Seemann T. Prokka: rapid prokaryotic genome annotation. Bioinformatics 2014;30:2068–2069.

22. Bolger AM, Lohse M, Usadel B. Trimmomatic: a flexible trimmer for Illumina sequence data. Bioinformatics 2014;30:2114–2120.

23. Kopylova E, Noé L, Touzet H. SortMeRNA: fast and accurate filtering of ribosomal RNAs in metatranscriptomic data. Bioinformatics 2012;28:3211–3217.

24. Langmead B, Salzberg SL. Fast gapped-read alignment with Bowtie 2. Nat Methods 2012;9:357–359.

25. Csardi G, Nepusz T. The igraph software package for complex network research. InterJournal, Complex Syst 2006;1695:1–9.

26. Smoot ME, Ono K, Ruscheinski J, Wang P-L, Ideker T. Cytoscape 2.8: new features for data integration and network visualization. Bioinformatics 2011;27:431–432.

27. Shaffer M, Borton MA, McGivern BB, Zayed AA, La Rosa SL 0003 3527 8101, Solden LM, et al. DRAM for distilling microbial metabolism to automate the curation of microbiome function. Nucleic Acids Res 2020;48:8883–8900.

28. Li W, Godzik A. Cd-hit: a fast program for clustering and comparing large sets of protein or nucleotide sequences. Bioinformatics 2006;22:1658–1659.

29. Fu L, Niu B, Zhu Z, Wu S, Li W. CD-HIT: accelerated for clustering the next-generation sequencing data. Bioinformatics 2012;28:3150–3152.

30. Parks DH, Chuvochina M, Waite DW, Rinke C, Skarshewski A, Chaumeil PA, et al. A standardized bacterial taxonomy based on genome phylogeny substantially revises the tree of life. Nat Biotechnol 2018;36:996.

31. Parks DH, Chuvochina M, Chaumeil P-A, Rinke C, Mussig AJ, Hugenholtz P. A complete domain-to-species taxonomy for Bacteria and Archaea. Nat Biotechnol 2020;38:1079–1086.

32. Chaumeil P-A, Mussig AJ, Hugenholtz P, Parks DH. GTDB-Tk: a toolkit to classify genomes with the Genome Taxonomy Database. Bioinformatics 2019;36:1925–1927.

33. Nguyen L-T, Schmidt HA, von Haeseler A, Minh BQ. IQ-TREE: A fast and effective stochastic algorithm for estimating maximum-likelihood phylogenies. Mol Biol Evol 2014;32:268–274.

34. Guindon S, Dufayard J-F, Lefort V, Anisimova M, Hordijk W, Gascuel O. New algorithms and methods to estimate maximum-likelihood phylogenies: assessing the performance of PhyML 3.0. Syst Biol 2010;59:307–321.

35. Rodriguez-R LM, Konstantinidis K. Bypassing cultivation to identify bacterial species: Culture-independent genomic approaches identify credibly distinct clusters, avoid cultivation bias, and provide true insights into microbial species. Microbe Mag 2014;9:111–118.

36. Ludwig W, Strunk O, Westram R, Richter L, Meier H, Yadhukumar, et al. ARB: a software environment for sequence data. Nucleic Acids Res 2004;32:1363–1371.

37. Konstantinidis KT, Rosselló-Móra R, Amann R. Uncultivated microbes in need of their own

taxonomy. ISME J 2017;11:2399–2406.

38. Herber J, Klotz F, Frommeyer B, Weis S, Straile D, Kolar A, et al. A single Thaumarchaeon drives nitrification in deep oligotrophic Lake Constance. Environ Microbiol 2020;22:212–228.

39. Spasov E, Tsuji JM, Hug LA, Doxey AC, Sauder LA, Parker WJ, et al. High functional diversity among *Nitrospira* populations that dominate rotating biological contactor microbial communities in a municipal wastewater treatment plant. ISME J 2020;14:1857–1872.

40. Sakoula D, Koch H, Frank J, Jetten MSM, van Kessel MAHJ, Lücker S. Enrichment and physiological characterization of a novel comammox *Nitrospira* indicates ammonium inhibition of complete nitrification. ISME J 2021;15:1010–1024.

41. Daims H, Lebedeva E V., Pjevac P, Han P, Herbold C, Albertsen M, et al. Complete nitrification by *Nitrospira* bacteria. Nature 2015;528:504–509.

42. Pjevac P, Schauberger C, Poghosyan L, Herbold CW, van Kessel MAHJ, Daebeler A, et al. *AmoA*-targeted polymerase chain reaction primers for the specific detection and quantification of comammox *Nitrospira* in the environment. Front Microbiol 2017;8:1–11.

43. Van Kessel MAHJ, Speth DR, Albertsen M, Nielsen PH, Op Den Camp HJM, Kartal B, et al. Complete nitrification by a single microorganism. Nature 2015;528:555–559.

Supplementary Tables

Table S1. Overview of metagenome-assembled genomes (MAGs) and contigs related to the nitrifying community in the hypolimnion of Lake Constance.

Table S2. Annotation and seasonally resolved transcription of MAG AOA-LC4 genes involved in nitrogen metabolism, vitamin synthesis, carbon fixation, cell division, replication, transport systems, respiration and the TCA-cycle.

Table S3. NCBI accession numbers or Taxon IDs of all species, MAGs and clones, which are part of the phylogenetic trees of *Nitrososphaeria*, *Nitrosomonadaceae* and *Nitrospira* (Figures 2, S3, S5, S6, S8, and S9).

Supplementary Figures

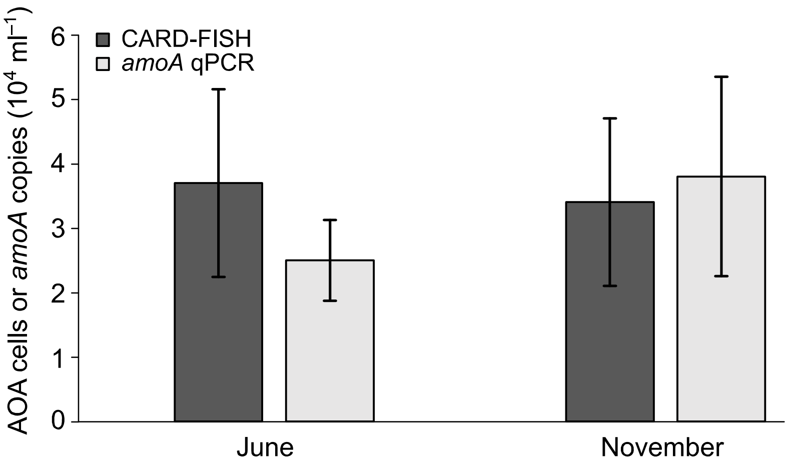

Figure S1. AOA abundance in hypolimnetic water from 85 m depth as measured by archaeal *amoA*-targeted qPCR or CARD-FISH using a *Nitrososphaeria*-specific probe (probe Thaum726), which currently encompasses all AOA. Samples were taken on June 18th and November 5th 2019. CARD-FISH was performed on water samples used for nitrification rate measurements after 67 h (June) or 48 h (November) of incubation at 4°C in the dark. Hybridized filters were counted either 10 times (June) or 25 times (November) independently.

ANI

	Nitrosopumilus sp. BACL13 MAG-120910-bin56	*Nitrosopumilus* sp. BACL13 MAG-121220-bin23	*Nitrosopumilus* sp. UBA241	*Nitrosopumilus* sp. Baikal-deep-G182	*Nitrosopumilus* sp. Casp-thauma1	**AOA-LC4**	*Nitrosopumilus* sp. Nsub	*Candidatus* Nitrosomarinus catalina SPOT01	*Nitrosopumilus* sp. MED-G94	*Candidatus* Nitrosarchaeum koreense MY1	*Candidatus* Nitrosarchaeum limniae BG20	*Candidatus* Nitrosarchaeum limniae SFB1	*Nitrosopumilus maritimus* SCM1	*Candidatus* Nitrosopumilus koreense AR1	*Nitrosopumilus piranensis* D3C	*Nitrosopumilus adriaticus* NF5	*Candidatus* Nitrosopumilus sediminis AR2	*Candidatus* Nitrosopumilus salarius BD31
Nitrosopumilus sp. BACL13 MAG-120910-bin56	100	100	100	86	86	86	84	85	85	79	79	79	80	80	80	81	81	81
Nitrosopumilus sp. BACL13 MAG-121220-bin23	100	100	100	85	85	85	83	84	84	78	78	78	79	79	79	80	80	79
Nitrosopumilus sp. UBA241	100	100	100	85	85	85	83	84	84	78	78	78	79	79	79	80	80	79
Nitrosopumilus sp. Baikal-deep-G182	86	85	85	100	99	99	84	84	84	77	77	78	78	78	79	79	79	79
Nitrosopumilus sp. Casp-thauma1	86	85	85	99	100	99	84	84	84	78	77	77	79	79	79	79	79	79
**AOA-LC4**	86	85	85	99	99	100	84	84	84	78	78	78	78	79	79	79	80	80
Nitrosopumilus sp. Nsub	84	83	83	84	84	84	100	89	89	77	78	78	79	79	79	79	80	79
Candidatus Nitrosomarinus catalina SPOT01	85	84	84	84	84	84	89	100	93	78	78	78	79	79	79	79	80	79
Nitrosopumilus sp. MED-G94	85	84	84	84	84	84	89	93	100	78	78	78	80	79	79	80	80	80
Candidatus Nitrosarchaeum koreense MY1	79	78	78	77	78	78	77	78	78	100	82	82	78	78	78	78	78	78
Candidatus Nitrosarchaeum limniae BG20	79	78	78	77	77	78	78	78	78	82	100	96	78	78	78	78	78	78
Candidatus Nitrosarchaeum limniae SFB1	79	78	78	78	77	78	78	78	78	82	96	100	78	78	78	78	78	78
Nitrosopumilus maritimus SCM1	80	79	79	78	79	78	79	79	80	78	78	78	100	85	85	80	80	80
Candidatus Nitrosopumilus koreense AR1	80	79	79	78	79	79	79	79	79	78	78	78	85	100	86	80	80	80
Nitrosopumilus piranensis D3C	80	79	79	79	79	79	79	79	79	78	78	78	85	86	100	80	80	80
Nitrosopumilus adriaticus NF5	81	80	80	79	79	79	79	79	80	78	78	78	80	80	80	100	81	81
Candidatus Nitrosopumilus sediminis AR2	81	80	80	79	79	80	80	80	80	78	78	78	80	80	80	81	100	82
Candidatus Nitrosopumilus salarius BD31	81	79	79	79	79	80	79	79	80	78	78	78	80	80	80	81	82	100

Colour scale: 100, 95, 90, 85, 80

AAI

	Nitrosopumilus sp. BACL13 MAG-120910-bin56	*Nitrosopumilus* sp. BACL13 MAG-121220-bin23	*Nitrosopumilus* sp. UBA241	*Nitrosopumilus* sp. Baikal-deep-G182	*Nitrosopumilus* sp. Casp-thauma1	**AOA-LC4**	*Nitrosopumilus* sp. Nsub	*Candidatus* Nitrosomarinus catalina SPOT01	*Nitrosopumilus* sp. MED-G94	*Candidatus* Nitrosarchaeum koreense MY1	*Candidatus* Nitrosarchaeum limniae BG20	*Candidatus* Nitrosarchaeum limniae SFB1	*Nitrosopumilus maritimus* SCM1	*Candidatus* Nitrosopumilus koreense AR1	*Nitrosopumilus piranensis* D3C	*Nitrosopumilus adriaticus* NF5	*Candidatus* Nitrosopumilus sediminis AR2	*Candidatus* Nitrosopumilus salarius BD31
Nitrosopumilus sp. BACL13 MAG-120910-bin56	100	98	98	84	84	85	85	86	86	75	75	75	78	79	79	80	80	78
Nitrosopumilus sp. BACL13 MAG-121220-bin23	98	100	100	84	84	84	83	84	85	73	73	73	77	77	77	77	78	77
Nitrosopumilus sp. UBA241	98	100	100	84	84	84	83	84	84	73	72	73	76	77	76	77	78	77
Nitrosopumilus sp. Baikal-deep-G182	84	84	84	100	99	99	85	85	85	74	74	74	77	77	77	77	78	77
Nitrosopumilus sp. Casp-thauma1	84	84	84	99	100	99	84	85	85	74	73	73	77	77	77	77	78	77
**AOA-LC4**	85	84	84	99	99	100	84	85	85	74	73	73	77	77	77	77	78	77
Nitrosopumilus sp. Nsub	85	83	83	85	84	84	100	92	92	73	73	72	77	77	77	77	78	76
Candidatus Nitrosomarinus catalina SPOT01	86	84	84	85	85	85	92	100	95	73	73	73	77	77	77	77	78	77
Nitrosopumilus sp. MED-G94	86	85	84	85	85	85	92	95	100	74	73	74	78	78	78	78	79	77
Candidatus Nitrosarchaeum koreense MY1	75	73	73	74	74	74	73	73	74	100	83	83	74	75	75	75	75	75
Candidatus Nitrosarchaeum limniae BG20	75	73	72	74	73	73	73	73	73	83	100	96	74	74	73	74	74	75
Candidatus Nitrosarchaeum limniae SFB1	75	73	73	74	73	73	72	73	74	83	96	100	74	74	74	74	74	74
Nitrosopumilus maritimus SCM1	78	77	76	77	77	77	77	77	78	74	74	74	100	87	87	80	80	79
Candidatus Nitrosopumilus koreense AR1	79	77	77	77	77	77	77	77	78	75	74	74	87	100	88	80	80	79
Nitrosopumilus piranensis D3C	79	77	76	77	77	77	77	77	78	75	73	74	87	88	100	80	80	79
Nitrosopumilus adriaticus NF5	80	77	77	77	77	77	77	77	78	75	74	74	80	80	80	100	81	80
Candidatus Nitrosopumilus sediminis AR2	80	78	78	78	78	78	78	78	79	75	74	74	80	80	80	81	100	81
Candidatus Nitrosopumilus salarius BD31	78	77	77	77	77	77	76	77	77	75	75	74	79	79	79	80	81	100

Colour scale: 100, 95, 90, 85, 80, 75

Figure S2. Pairwise genome-wide average nucleotide identities (ANI) and average amino acid identities (AAI) of MAG AOA-LC4 (shown in bold) in comparison to representatives of the family *Nitrosopumilaceae*. MAG AOA-LC4 represents a novel species together with Casp-thauma1 and

Baikal-Deep-G182 in the genus *Nitrosopumilus* based on the species-level threshold of 95% for ANI [37] and genus-threshold of 65% for AAI [37].

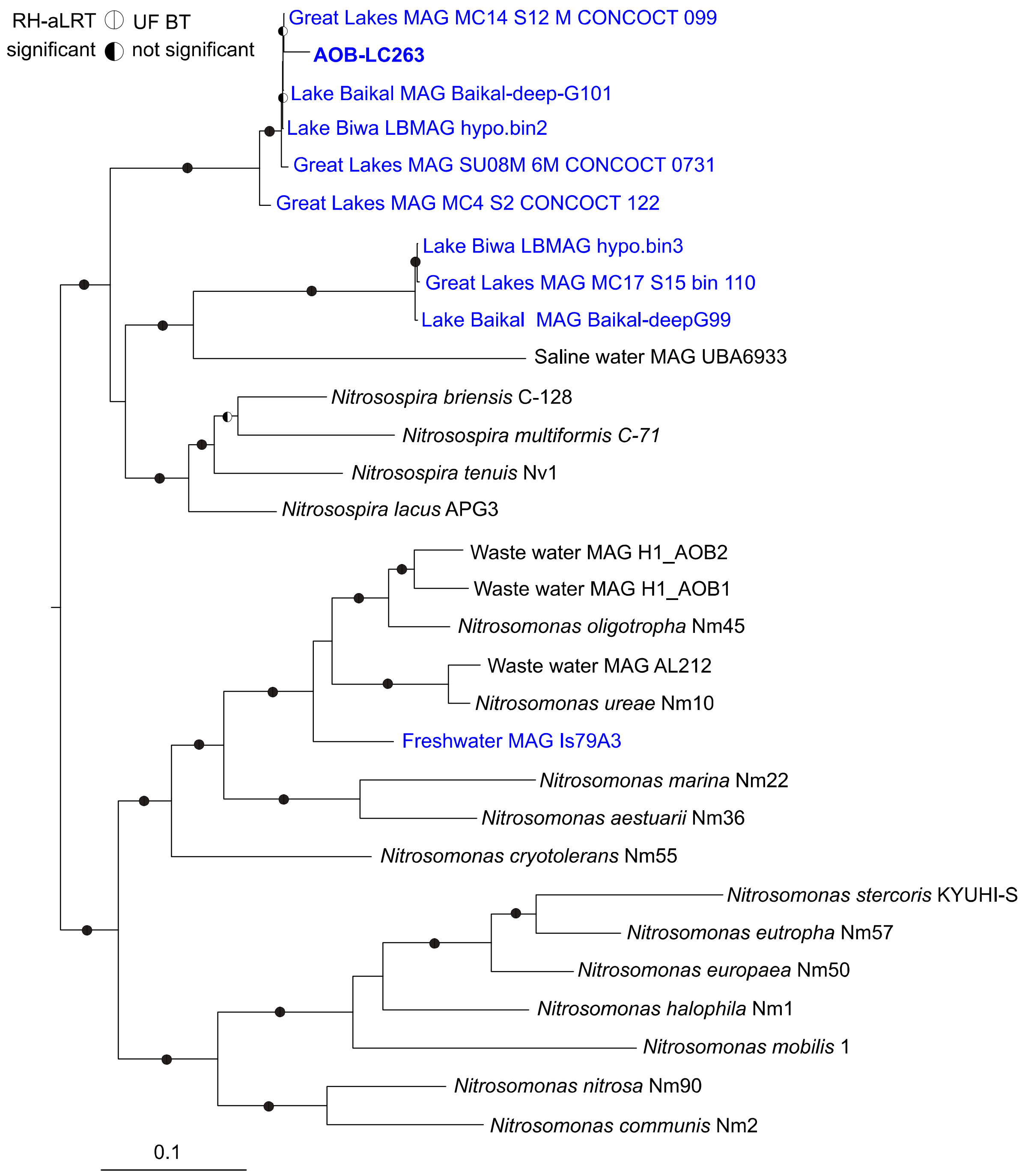

Figure S3. Phylogeny of MAG AOB-LC263 in relation to closely related freshwater MAGs and pure cultures of the ammonia-oxidizing bacteria within the genera *Nitrosomonas* and *Nitrosospira*. The phylogenomic maximum likelihood tree was constructed using the IQ-tree algorithm [33] on the basis of a concatenated amino acid alignment of 120 translated single copy genes that were established by the GTDB-based taxonomy for phylogenetic inference of bacteria [30, 31]. Branch

support was tested with the Shimodaira–Hasegawa approximate likelihood-ratio test (SH-aLRT; 1000 replicates) and ultrafast bootstraps (1000 replicates) within IQ-tree. Branch support was set as significant at ≥80% for SH-aLRT and ≥95% for ultrafast bootstrap values (black semi-circles for significant and white for non-significant). MAGs or species with freshwater-origin are colored blue. *Methylotenera mobilis* (NCBI accession number GCA_000023705.1), *Methylovorus glucosetrophus* (NC_012969.1) and *Methylobacillus flagellates* (GCA_000013705.1) were used as outgroup. The scale bar indicates 10% estimated amino acid sequence divergence. All accession numbers can be found in Table S3.

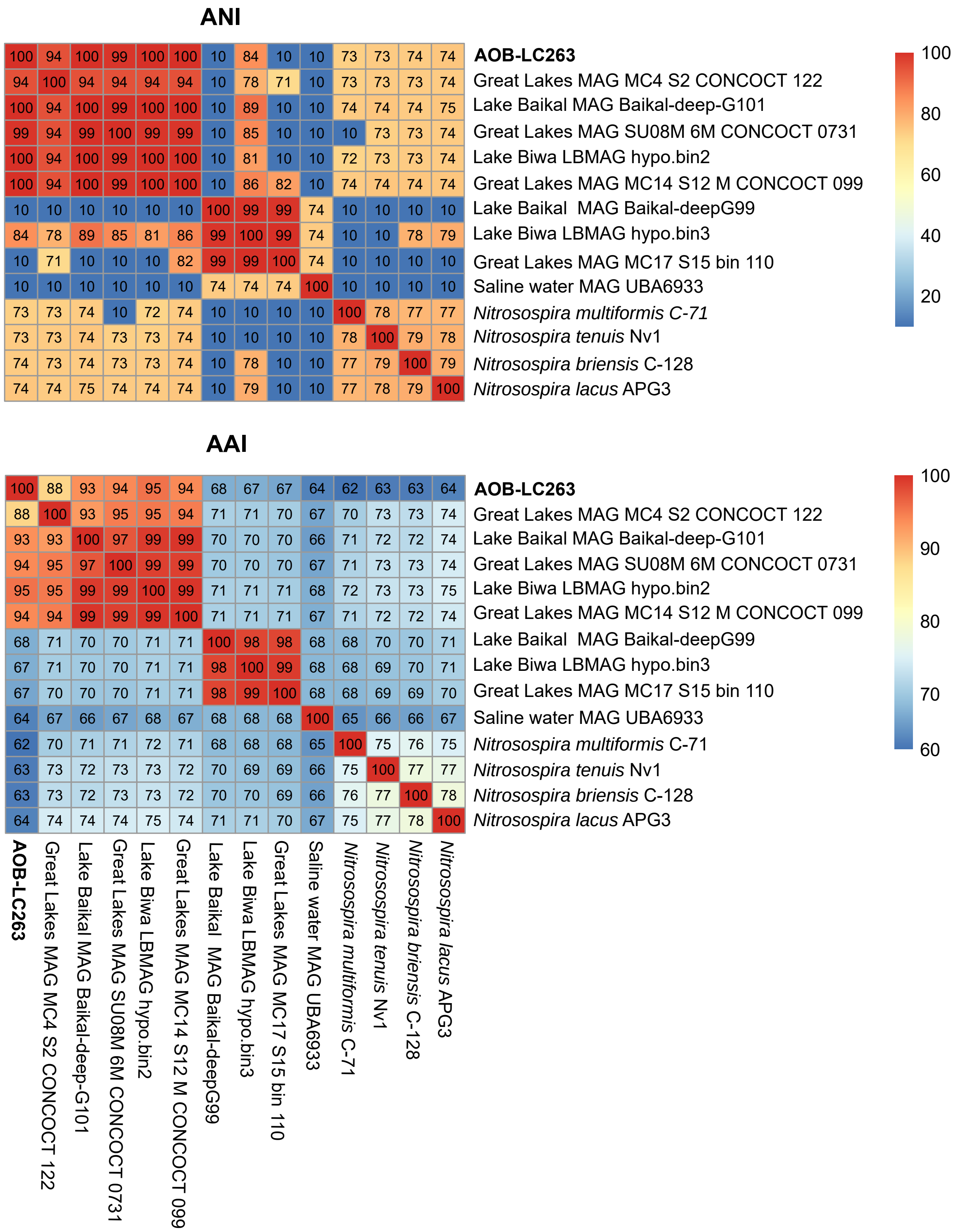

Figure S4. Pairwise genome-wide average nucleotide identities (ANI) and average amino acid (AAI) identities for MAG AOB-LC263 (shown in bold) in comparison to closely related MAGs and representatives of the genus *Nitrosospira*. AOB-LC263 represents a novel genus compared to described *Nitrosospira* species based on the threshold of 65% for AAI [37].

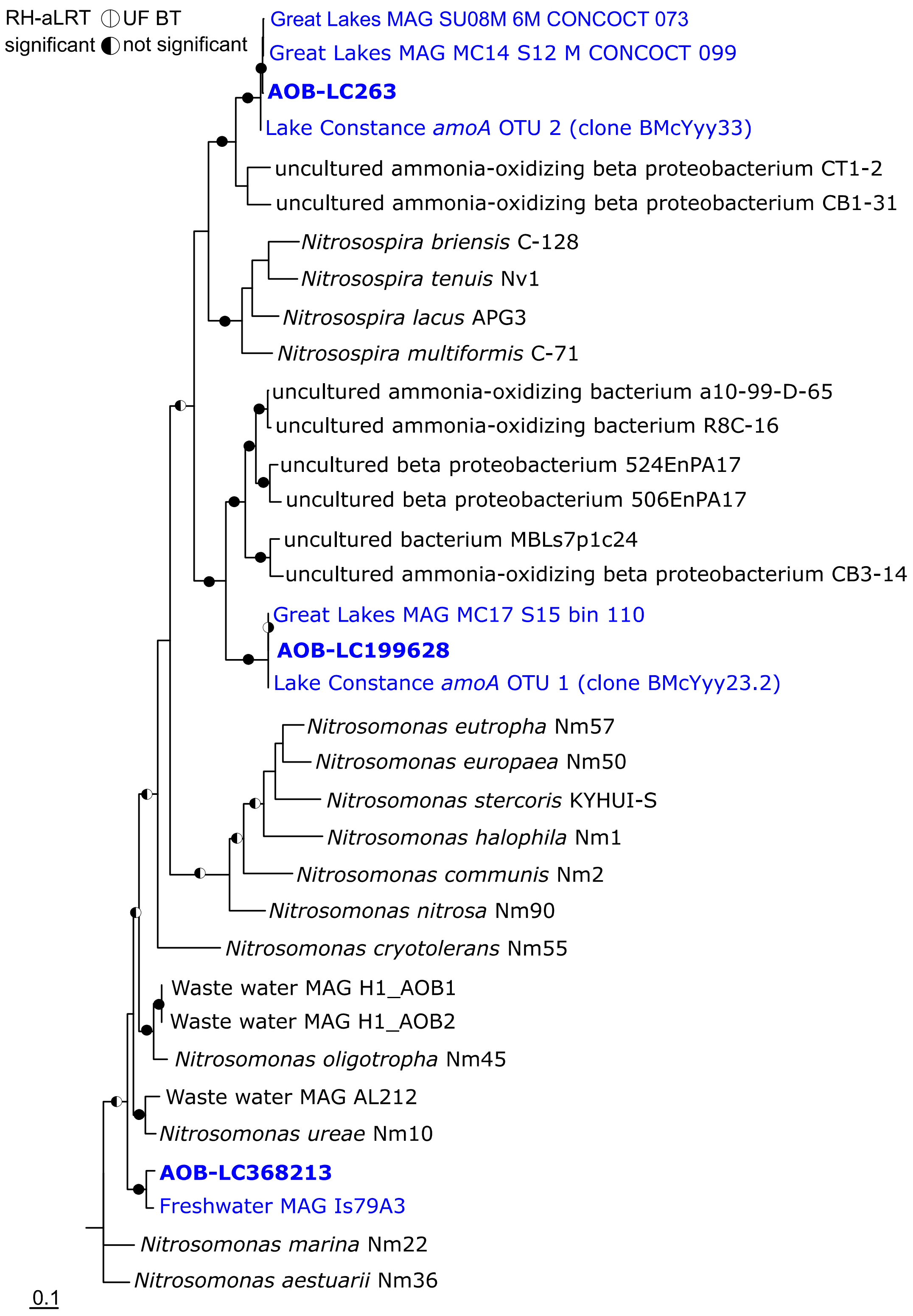

Figure S5. Phylogeny of MAG AOB-LC263 and contigs AOB-LC199628 and AOB-LC368213 in relation to ammonia-oxidizing bacteria and environmental sequences affiliated with the family *Nitrosomonadaceae* as based on the functional marker gene *amoA*. The maximum likelihood tree was inferred by the IQ-tree algorithm [33] using 459 unambiguous alignment positions of the bacterial *amoA* gene. Branch support was tested with the Shimodaira–Hasegawa approximate likelihood-ratio test (SH-aLRT; 1000 replicates) and ultrafast bootstraps (1000 replicates) within IQ-

tree. Branch support was set as significant at ≥80% for SH-aLRT and ≥95% for ultrafast bootstrap values (black semi-circles for significant and white for non-significant). MAGs, clones or species with freshwater-origin are colored blue. *Nitrosococcus watsonii* (NC_014315) and *Nitrosococcus oceani* (NC_007484) *amoA* genes were used as outgroup. The scale bar indicates 10% estimated nucleic acid sequence divergence. Accession numbers can be found in Table S3.

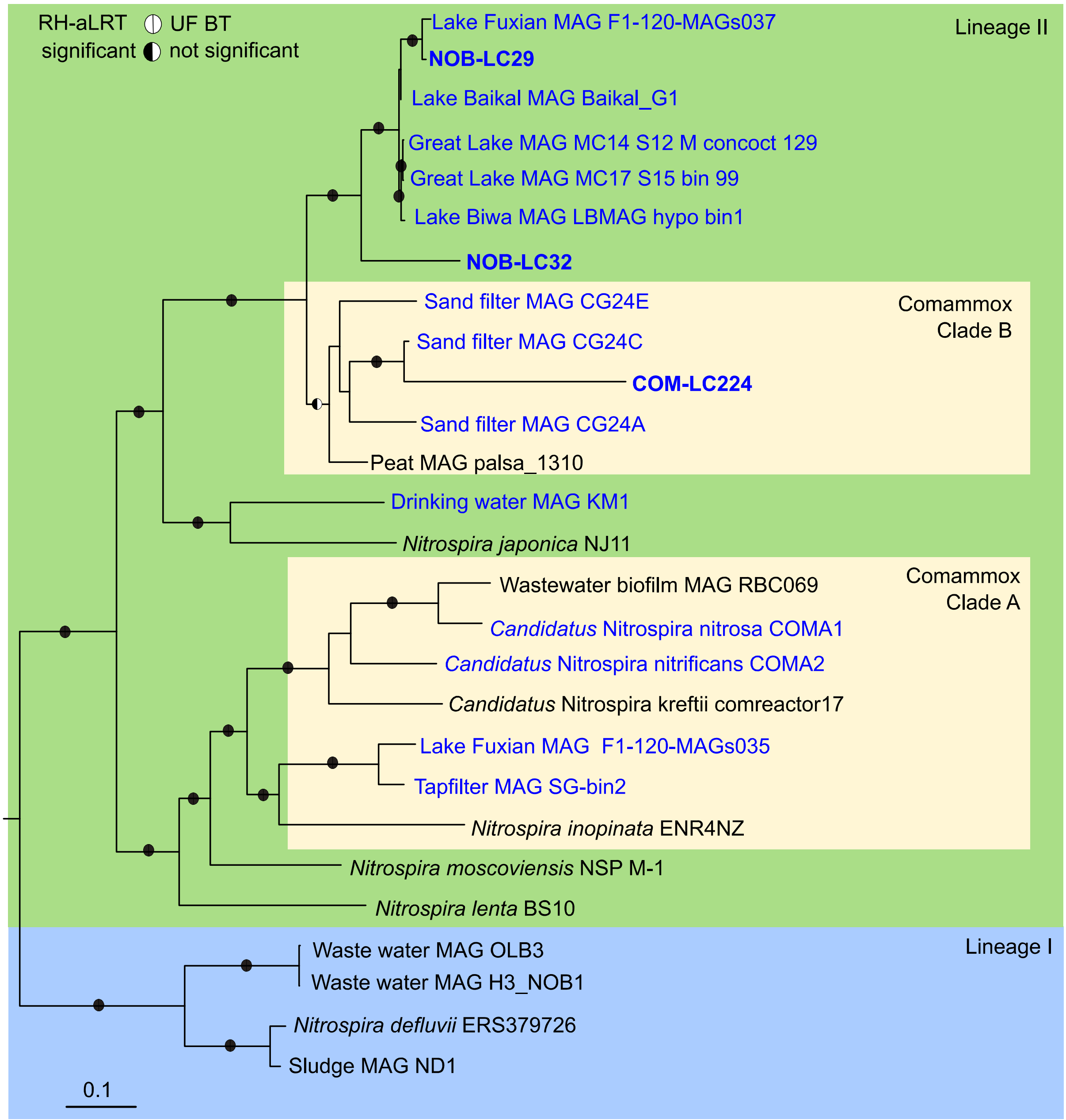

Figure S6. Phylogeny of MAGs NOB-LC29, NOB-LC32, and COM-LC224 in relation to representatives of *Nitrospira* lineage I and II. MAGs affiliated with either comammox clade A or B were taken from the literature [39, 40]. Clade classification of comammox bacteria is based on their *amoA* gene as proposed by Daims *et al.* [41] and Pjevac *et al*. [42]. The phylogenomic maximum likelihood tree was constructed using the IQ-Tree algorithm [33] on the basis of a concatenated amino acid alignment of 120 translated single copy genes that were established by the GTDB-based taxonomy for phylogenetic inference of bacteria [30, 31]. Branch support was tested with the Shimodaira–Hasegawa approximate likelihood-ratio test (SH-aLRT; 1000 replicates) and ultrafast bootstraps (1000 replicates) within IQ-tree. Branch support was set as significant at ≥80% for SH-aLRT and ≥95% for ultrafast bootstrap values (black semi-circles for significant and white for non-significant). MAGs or

species with freshwater-origin are colored blue. *Leptospirillum ferriphilum* (GCA_900198525.1) and *Leptospirillum ferrooxidans* (GCA_000284315.1) were used as outgroup. The scale bar indicates 10% estimated amino acid sequence divergence. Accession numbers can be found in Table S3.

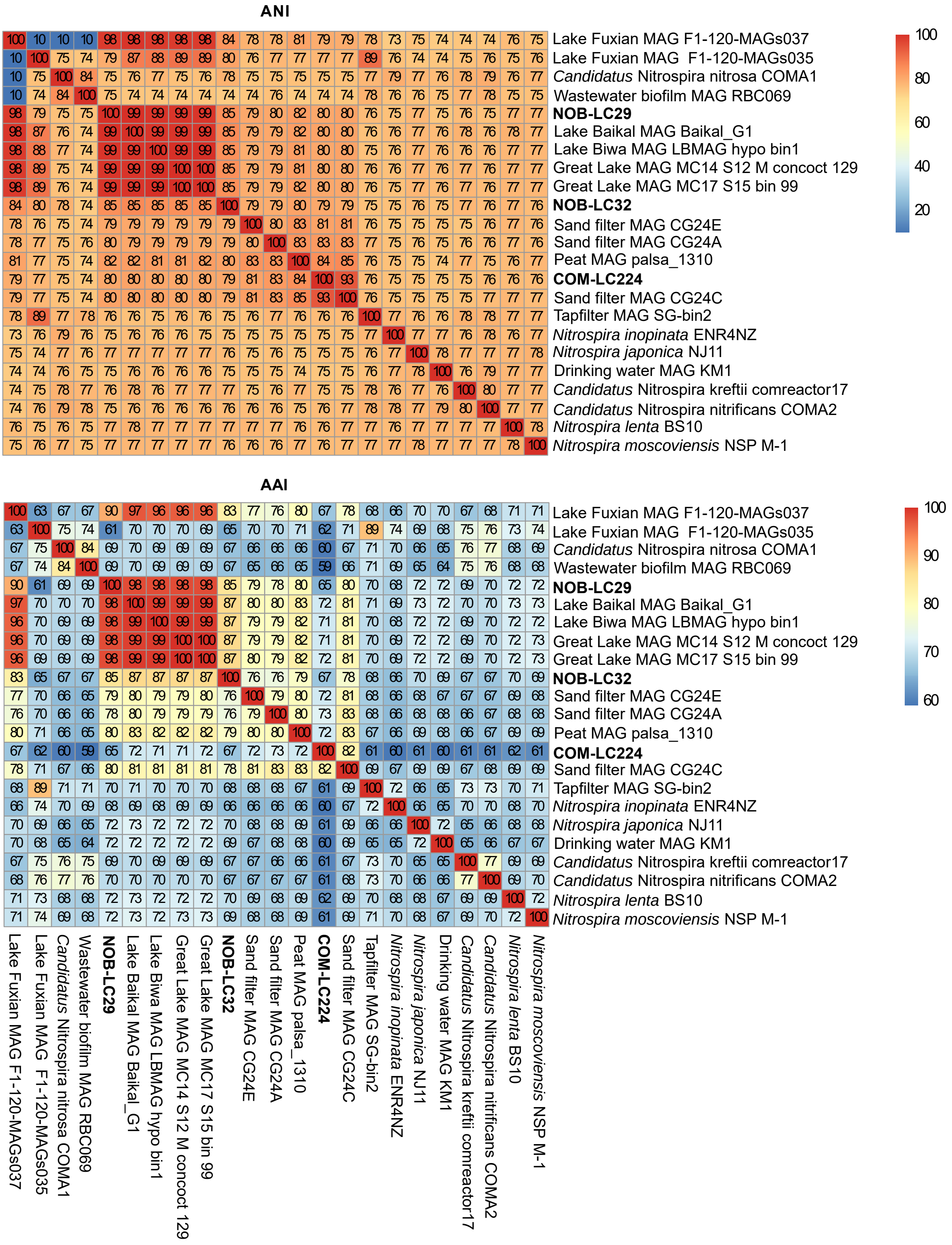

Figure S7. Pairwise genome-wide average nucleotide identities (ANI) and average amino acid identities (AAI) for lineage II *Nitrospira* including MAGs NOB-LC32, NOB-LC29, and COM-LC224 (shown in bold).

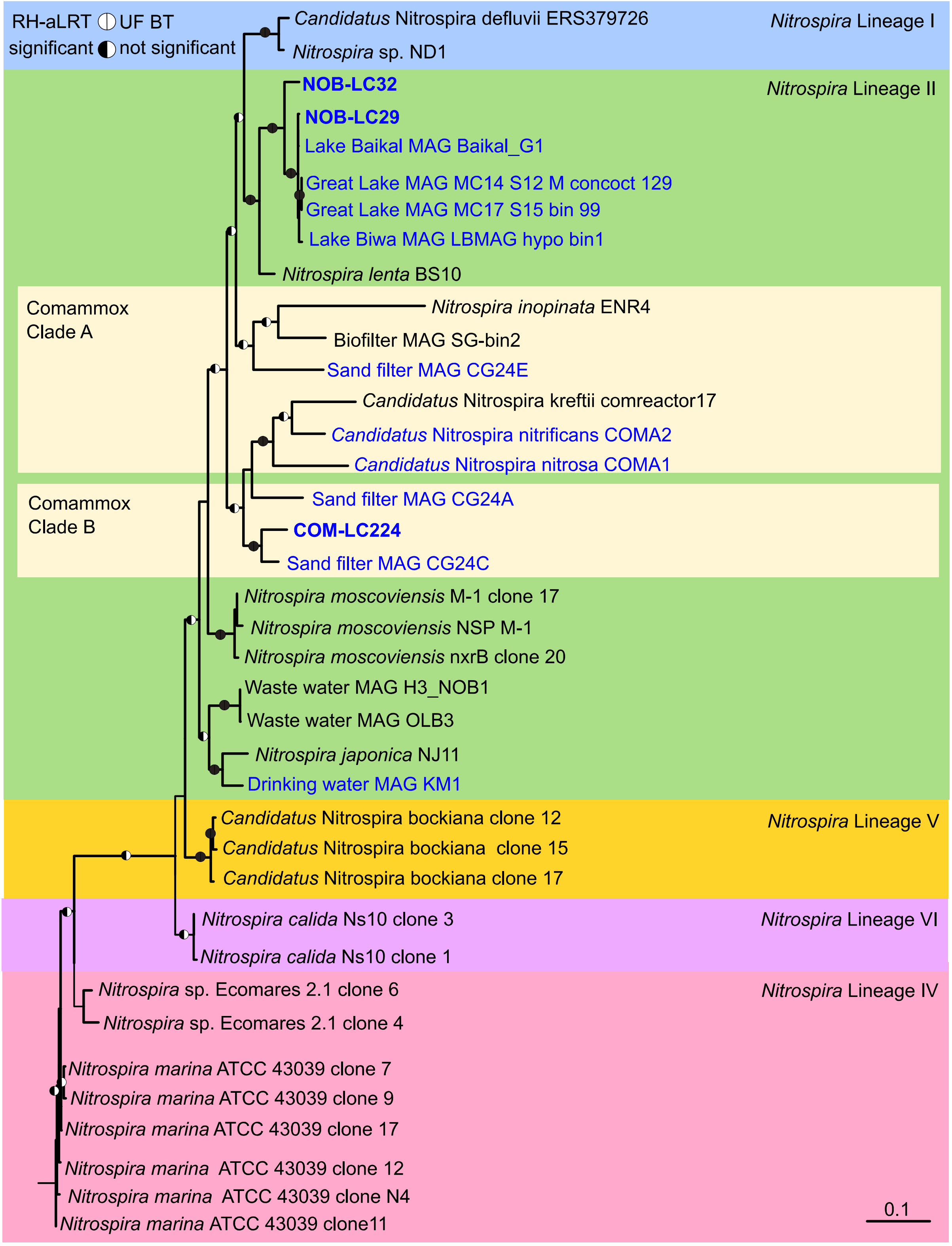

Figure S8. Phylogeny of MAGs NOB-LC29, NOB-LC32, and COM-LC224 as based on the functional marker gene *nxrB*. Classification of the comammox MAGs into clade A or B was transferred from the phylogenomic tree (Fig. S6), as comammox species cannot be distinguished based on their *nxrB* gene from canonical *Nitrospira* species [41, 43]. The maximum likelihood tree was inferred by the IQ-tree algorithm [33] using 1,205 unambiguous alignment positions of the *nxrB* gene. Branch support was tested with the Shimodaira–Hasegawa approximate likelihood-ratio test (SH-aLRT; 1000 replicates)

and ultrafast bootstrap (1000 replicates) within the IQ-tree software package. Branch support was set as significant at ≥80% for SH-aLRT and ≥95% for ultrafast bootstrap values (black semi-circles for significant and white for non-significant). MAGs, clones or species with freshwater-origin are colored blue. *Hydrogenobaculum* sp. (NC_011126), *Natronomonas pharaonis* (NC_007426.1) and *Candidatus* Kuenenia stuttgartiensis (CT573072) *narH*-like genes were used as outgroup. The scale bar indicates 10% estimated nucleic acid sequence divergence. Accession numbers can be found in Table S3.

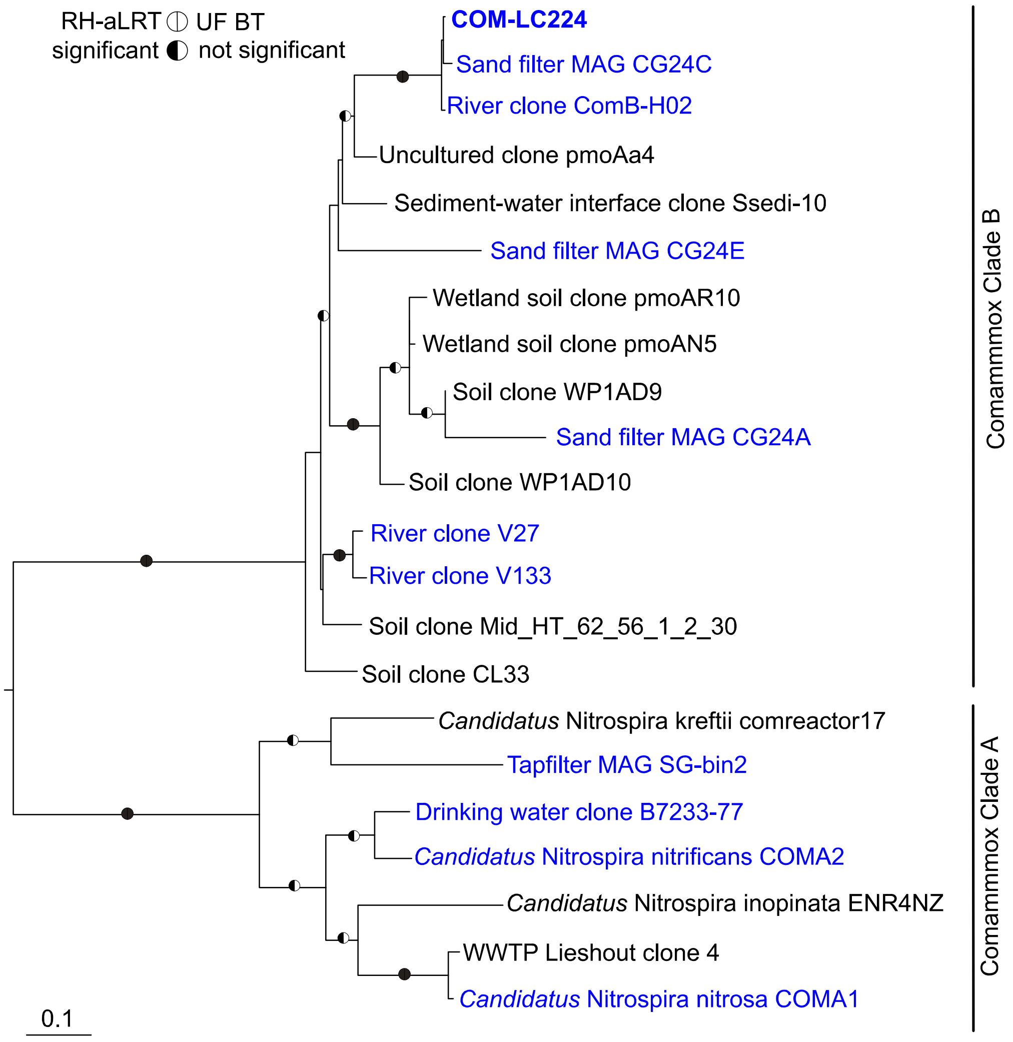

Figure S9. Phylogeny of COM-LC224 as based on the functional marker gene *amoA*. Classification into clade A or B was done as proposed by by Daims *et al.* [41] and Pjevac *et al.* [42]. The maximum likelihood tree was inferred by the IQ-tree algorithm [33] using 414 unambiguous alignment positions of the *amoA* gene. Branch support was tested with the Shimodaira–Hasegawa approximate likelihood-ratio test (SH-aLRT; 1000 replicates) and ultrafast bootstrap (1000 replicates) within the IQ-tree software package. Branch support was set as significant at ≥80% for SH-aLRT and ≥95% for ultrafast bootstrap values (black semi-circles for significant and white for non-significant). MAGs or species with freshwater-origin are colored blue. *Nitrosospira multiformis* (U91603), *Nitrosospira* sp. (WP_041514847.1*),* *Nitrosomonas europaea* (L08050) and *Nitrosomonas communis* (WP_046851395) *amoA* genes were used as outgroup. The scale bar indicates 10% estimated nucleic acid sequence divergence. Accession numbers can be found in Table S3.

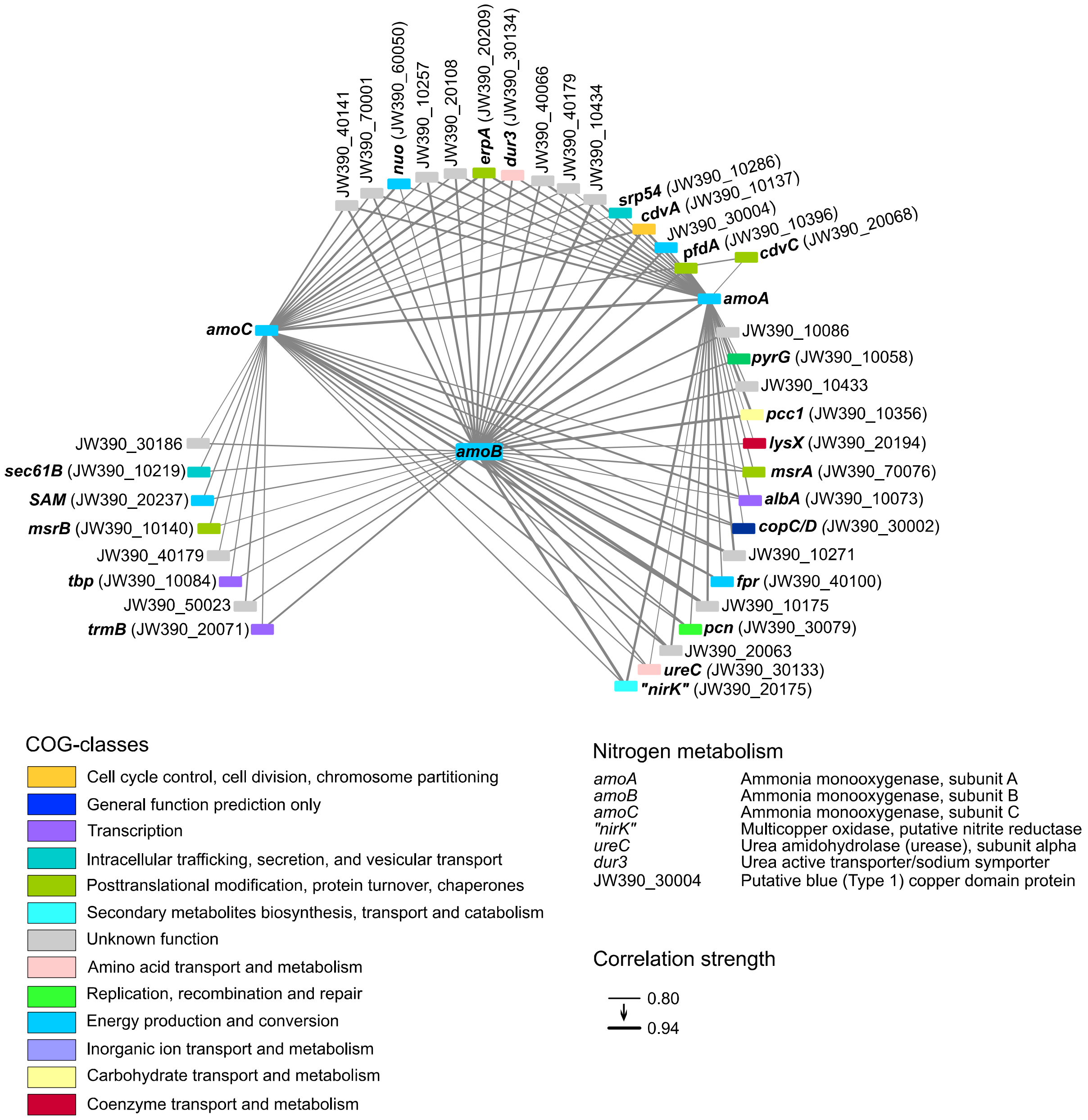

Figure S10. Network analysis of ammonia oxidation-related and co-transcribed AOA-LC4 genes over the yearly cycle. Only genes with a strong and significant (Spearman´s $r \geq 0.8$, FDR-corrected p-value <0.05) correlation to at least two of the *amoABC* genes and an average expression higher than the median of all transcribed genes were considered for the network analysis. Correlation coefficient´s strength is visualized by increasing width of the edges. Detailed gene annotation and transcription values can be found in Table S2.

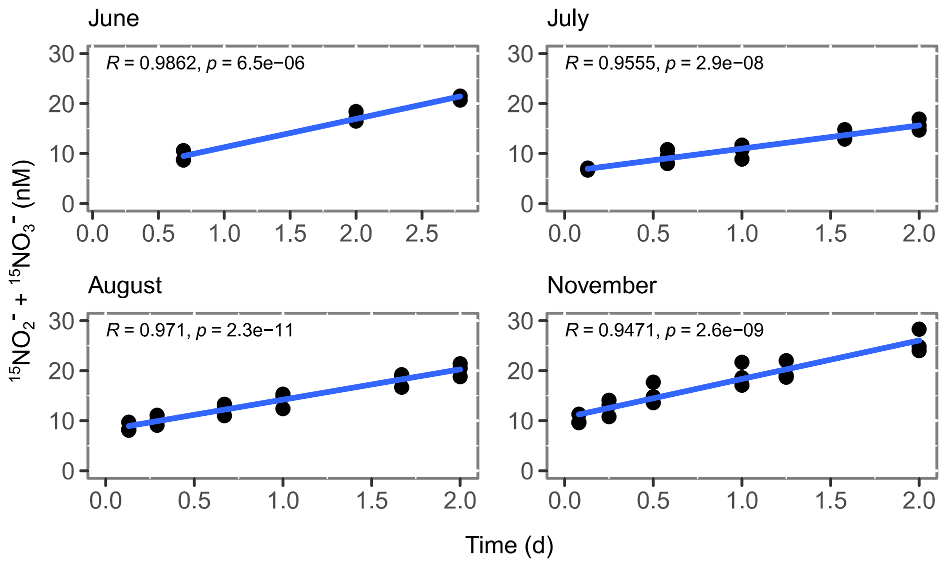

Figure S11. ^{15}N-nitrite/nitrate production from ^{15}N-ammonium in incubations of hypolimnetic water taken from 85 m depth. Produced ^{15}N-nitrite/nitrate is shown in relation to the first sampling time point. Linear regression through the time points was used to infer ammonia oxidation rates. All incubations were performed in biological triplicates at 4°C in the dark over a period of 48 h, except for June with 67 h. Samples were taken in 2019 on June 18th, July 29th, August 28th and November 5th. Incubations started within 1−7 h after sampling.
